# Direct reprogramming of Müller glia into photoreceptors via multiple transcription factors and small molecules: molecular mechanisms and transcriptomic analysis

**DOI:** 10.1007/s11626-026-01164-0

**Published:** 2026-02-25

**Authors:** Yuka Endo, Eriko Sugano, Yuko Seko, Tomokazu Fukuda, Kitako Tabata, Taira Kakizaki, Shu Maruoka, Takanori Yokoyama, Hanaho Mori, Taku Ozaki, Lanlan Bai, Hiroshi Tomita

**Affiliations:** 1 Laboratory of Visual Neuroscience, Molecular Medical Science, Iwate University Division of Agriculture, 4-3-5 Ueda, Morioka, Iwate 020-8551 Japan; 2 Sensory Functions Section, Research Institute, National Rehabilitation Center for Persons With Disabilities, 4-1 Namiki, Tokorozawa, 359-8555 Japan; 3 Laboratory of Cell Engineering and Molecular Genetics, Molecular Medical Science, Iwate University Division of Agriculture, 4-3-5 Ueda, Morioka, Iwate 020-8551 Japan; 4 Laboratory of Cell Biochemistry, Department of Life Sciences, Faculty of Agriculture, Iwate University, 3-18-8 Ueda, Morioka, Iwate 020-8550 Japan

**Keywords:** Direct reprogramming, Müller glia, Photoreceptor, Transcriptional factor, Polycistronic expression

## Abstract

Direct reprogramming, involving overexpression of transcription factors in combination with specific small-molecule treatments to induce cell fate conversion, represents a promising strategy for regenerative medicine and disease modeling. Monocistronic expression of four transcription factors (*CRX*,* NEUROD*,* RAX*, and *OTX2*) has been demonstrated to induce the expression of phototransduction-associated genes and confer light responsiveness in fibroblasts. In contrast, polycistronic expression of the same four factors resulted in the upregulation of only two cone-specific genes, indicating that optimization of reprogramming conditions is required to achieve complete photoreceptor differentiation. In this study, we focused on Müller glial cells, which exhibit regenerative potential in the retina of lower vertebrates, and explored their reprogramming into photoreceptor-like cells. We thus combined polycistronic expression of the aforementioned transcription factors and a pharmacological reprogramming approach utilizing a defined cocktail of small molecules. Transcriptomic analysis revealed that transcription factor introduction alone led to the upregulation of the genes related to neuronal identity and extracellular matrix components. Notably, the combination of transcription factor expression and chemical treatment induced photoreceptor-specific gene expression. However, no genes associated with phototransduction were detected, suggesting that further refinement is required to promote full differentiation into functionally mature light-responsive photoreceptors. Overall, our findings demonstrate that immortalized Müller glia can partially activate neuronal and photoreceptor genes through reprogramming, suggesting their potential for scalable drug screening and disease modeling. Clinical trial number: not applicable.

**Supplementary information:**

The online version contains supplementary material available at 10.1007/s11626-026-01164-0.

## Introduction

Müller glia in teleost fish, like zebrafish, potentially have the characteristics of retinal stem cells, which play a role in the self-repair system in response to retinal injury (Goldman 2014). In newts, even when the neural retina is removed from the eye, *Pax6* expression in retinal pigment epithelial cells can reprogram them into retinal neurons and regenerate a functional retina (Casco-Robles *et al*. 2016).

However, in mammals, these responses are insufficient for repairing retinal damage. Therefore, photoreceptor degeneration caused by progressive retinal diseases, such as retinitis pigmentosa and age-related macular degeneration, leads to irreversible vision loss. To prevent loss of photoreceptors from degeneration in retinitis pigmentosa and age-related macular degeneration, various treatments using chemicals such as antioxidants (Tomita *et al*. 2005) and ATPase inhibitors (Ikeda *et al*. 2014), cell transplantation of pluripotent stem cell-derived retinal pigment epithelial cells (Mandai *et al*. 2017), and gene therapy (Miyazaki *et al*. 2003) have been proposed. However, effective treatments for these diseases have not yet been established. Methods for restoring vision for blindness have also been investigated and are currently undergoing clinical trials (Jose Alain Sahel *et al*. 2022). Optogenetically mediated gene therapies are a promising treatment for restoring vision in patients with blindness (Bi *et al*. 2006; Watanabe *et al*. 2021). However, the visual function restored by optogenetically mediated gene therapies is inferior to native vision in terms of light and wavelength sensitivity (Tomita *et al*. 2014; Sato *et al*. 2017; Watanabe *et al*. 2018).

Direct reprogramming or direct conversion is used to change the direction of cell differentiation by transducing some transcriptional factors or exposing somatic cells to chemicals and microRNAs (Horisawa and Suzuki 2020). Using direct reprogramming technology, cells of interest can be easily and quickly reproduced without complete initialization of cells, such as in induced pluripotent stem cells. Various cell types have been generated using direct reprogramming. Vierbuchen *et al*. identified a combination of three neural lineage–specific transcription factors (*Ascl1*,* Brn2*, and *Myt1l*). These factors were introduced into mouse embryonic fibroblasts and reprogrammed into induced neuronal cells (Vierbuchen *et al*. 2010). Sekiya *et al*. introduced *Hnf4α* plus *Foxa1*,* Foxa2*, or *Foxa3* into mouse fibroblasts and reprogrammed them into induced hepatocyte-like (iHep) cells. iHep cells express multiple hepatocyte-specific markers and can reconstitute damaged hepatic tissues (Sekiya and Suzuki 2011). Ieda *et al*. transduced *Gata4*,* Mef2c*, and *Tbx5*, development-related transcriptional factors, into mouse cardiac fibroblasts and reprogrammed them into induced cardiomyocytes. The induced cardiomyocytes express cardiomyocyte-specific genes and generate spontaneous contractions and action potentials (Ieda *et al*. 2010).

Regarding photoreceptor cell generation, transduction of transcriptional factors *CRX* and *RAX*, which participate in retinal differentiation, and *NEUROD*, the transcriptional factor related to neuronal differentiation, into iris pigment epithelial cells, is reported to induce the expression of some photoreceptor-specific genes (Seko *et al*. 2012). Further, the expression level of photoreceptor-specific genes is increased by additional *OTX2* gene transduction in human dermal fibroblasts, and the reprogrammed cells can respond to light (Seko *et al*. 2014). Furthermore, in vivo studies have shown that the introduction of *Otx2*,* Crx*, and *Nrl* into Müller glia leads to the generation of Müller cell–derived rod photoreceptors in retinas of mice with congenital stationary night blindness, resulting in the partial restoration of visual function (Yao *et al*. 2018). These studies indicate that the transduction of multiple factors is required to generate photoreceptor cells.

However, Yao *et al*. employed a monocistronic expression system for the delivery of transcription factors into the retina, which may result in variability of factor expression among cells. To address this shortcoming, we previously constructed polycistronic vectors for four factors (*CRX*,* RAX*,* NEUROD*, and *OTX2*) and introduced them into human embryonic fibroblasts (Fukuda *et al*. 2018). This design allows the simultaneous delivery of all transcription factors into individual cells; this resulted in the upregulation of two photoreceptor-specific genes, *CNGB3* and *PDE6C*; however, other photoreceptor-specific genes *RHO*,* SAG*,* RCVRN*,* OPN1SW*, and *OPN1MW* were not upregulated. We also observed early neuronal cell markers and MAP2-positive cells, indicating that the reprogrammed cells remained in the early stages of the reprogramming process. Some studies have shown that some chemical compounds, including cell signaling inhibitors, improve the efficiency of differentiation into photoreceptors (Mahato *et al*. 2020; Fujii *et al*. 2023). Previous studies have mainly relied on monocistronic expression systems or pharmacological approaches for direct reprogramming into photoreceptors. However, the combined use of transcription-factor delivery via polycistronic vectors and small-molecule treatment remains largely unexplored. Furthermore, compounds used for reprogramming may exert distinct effects at the early differentiation and maturation stages, indicating that the optimization of the timing and conditions of compound addition could improve reprogramming efficiency. However, in studies using primary cultures, limitations in cell proliferation and batch-to-batch variability pose challenges for detailed screening. To address these issues, we aimed to analyze transcriptomic changes induced in immortalized Müller cells by introducing four factors (*CRX*,* RAX*,* NEUROD*, and *OTX2*) using a polycistronic expression system and exposing these cells to multiple chemical compounds. This approach enabled the development of a more efficient and rational method for inducing photoreceptor identity, with potential applications for in vivo reprogramming strategies and generation of cell models for drug screening.

## Materials and methods

### Institutional review board statement

This study did not involve human participants or laboratory animals. The recombinant DNA experiments were approved by the Iwate University Committee for recombinant DNA experiments.

### Cell culture

rMC-1 cells, immortalized rat retinal Müller cells transformed with the SV40 large T antigen, were obtained from Applied Biological Materials Inc (T0576; Richmond, Canada). rMC-1 cells were cultured in Dulbecco’s modified Eagle medium (DMEM; Thermo Fisher Scientific, Waltham, MA) supplemented with 10% FBS (Thermo Fisher Scientific), 1 × antibiotic-antimycotic solution (Thermo Fisher Scientific), 1 × GlutaMax (Thermo Fisher Scientific), and 0.35% D-glucose (FUJIFILM Wako Pure Chemical Corporation, Osaka, Japan), on culture plates coated with atelocollagen (KOKEN, Tokyo, Japan). 293 T cells, human embryonic kidney cells transformed with the SV40 large T antigen, were kindly provided by the RIKEN BioResource Center(Tsukuba, Japan). 293T cells were cultured in DMEM supplemented with 10% FBS and 1 × antibiotic-antimycotic solution. Both rMC-1 and 293 T cells were cultured at 37 °C under a 95% air:5% CO2 atmosphere.

### Construction and introduction of the hGRK1 reporter gene plasmid

A pCherry-expression vector driven by the human G protein-coupled receptor kinase1 (hGRK1) promoter was constructed using a pNL2․3 vector including a hygromycin resistance gene (Promega, Madison, WI). The hGRK1 promoter and pCherry were inserted into the multicloning site of the pNL2․3 Vector (pNL-GRK-Cherry). The sequence of pNL-GRK-Cherry was confirmed by Sanger sequencing (Genewiz, South Plainfield, NJ). The pNL-GRK-Cherry vector was linearized by restriction digestion and electroporated into rMC-1 cells. Electroporation was performed using an in vitro gene delivery system (NEPA GENE, Chiba, Japan) under conditions of 150 V, a pulse width of 10 ms, and a pulse interval of 50 ms. To isolate cells transduced with the pNL-GRK-Cherry vector, the cells were cultured in rMC-1 medium containing 800 ng/mL hygromycin (NACALAI TESQUE, INC, Kyoto, Japan) for 7 d.

### Production of the recombinant lentiviral vector

The plasmids including reprogramming factors (*CRX*,* NEUROD*,* RAX*, and *OTX2*) and eGFP genes (CSⅡ-CMV-CNROE) (Fukuda *et al*. 2018) were introduced into 293 T cells along with packaging plasmids (pcDNA3.1_TAR_VSVG_RSV-Rev, pcDNA3.1_TAR_TAT, MN1_TAR_HIVgp) to produce recombinant lentiviruses. Construction of these plasmids has been previously described. After transfection for 72 hours, the culture medium was changed to 293 T medium containing 10 µM forskolin (FUJIFILM Wako Pure Chemical Corporation), followed by additional incubation for 48 hours. After incubation, the supernatant containing lentivirus was collected. To remove cell debris, the supernatant was filtered using a 0.45-µm filter. The lentiviruses were then concentrated using a virus-concentration solution.

### Establishment of a stable CNROE expression cell line

rMC-1 cells transfected with pNL-GRK-Cherry were seeded in six-well plates at a density of 4 × 10^4^ cells/well. The cells were then exposed to the virus for 72 h. The lentivirus-infected cells were sorted for eGFP expression using a Cell Sorter SH800 (Sony, Tokyo, Japan). Next, to purify the stably transfected cell clones, the transfected cells were cloned using cloning cylinders. Independent transduction and single-cell cloning experiments were performed three times, resulting in three clonal lines. As controls, the cells were transduced with vectors encoding eGFP only (without transcription factors) and subjected to the same cloning procedure, yielding three control lines. For all subsequent experiments, rMC-1 cells at passages 42–55 (passages 18–25 after recombinant lentiviral transduction) were used.

### Differentiation into photoreceptors

CNROE-rMC-1 cells were seeded on a culture dish at a density of approximately 4.25 × 10^4^ cells/cm^2^, and eGFP-rMC-1 (control) cells were seeded at a density of approximately 4 × 10^4^ cells/cm^2^ in rMC-1 medium. The medium was changed to serum-free medium (SFM) containing 0.35% D-glucose, 1% GlutaMax, and 5 µg/mL fibronectin (Thermo Fisher Scientific) in DMEM on the next day after seeding. Thereafter, half of the SFM was replaced every other day. After 4 days, the medium was replaced with photoreceptor differentiation medium (PDM), and the cells were maintained in PDM for up to 4 wk. The PDM containing 0.35% D-glucose, 1% GlutaMax, fibronectin (5 µg/mL), 2% B27 Supplement (Thermo Fisher Scientific), Valproic acid (500 µM, Thermo Fisher Scientific), CHIR-99021 (4.8 µM, Cayman Chemical, Ann Arbor, MI), forskolin (10 µM, FUJIFILM Wako Pure Chemical Corporation), retinoic acid (500 nM, Sigma-Aldrich, St. Louis, MO), and DAPT (10 µM, Merck, Hessen, Germany) in DMEM. The PDM was completely replaced every other day.

### RT-PCR and RT-qPCR

Total RNA was extracted from cultured cells using the Reliaprep RNA Cell Miniprep System (Promega). cDNA was synthesized using the ReverTra Ace® qPCR RT Kit (TOYOBO, Osaka, Japan). KOD-FX (TOYOBO) was used for RT-PCR amplicon, and specific transcripts were amplified on the MJ Mini™ personal thermal cycler (Bio-Rad Laboratories, Hercules, CA). RT-qPCR was performed using the SsoAdvanced™ Universal SYBR® Green Supermix (Bio-Rad Laboratories, Tokyo, Japan); the primers used are listed in Table 1. RNA expression levels were quantified using the CFX Connect Real-Time PCR Analysis System (Bio-Rad Laboratories). Gapdh was used as the reference gene, and expression was quantified using the comparative Ct method. For each time point, expression levels were normalized to eGFP. The primers used in this study are listed in Table 1.

**Table 1.** Primer sequences for the RT-PCR and RT-qPCR in this study

Name	Sequence (5' → 3')	Product	Annealing
		(bp)	Temp (°C)
CRX	F:TCCGGCCCTAGCTTGACTAG	111	60
	R:GAGGCCTGAGAAGTAGGAGGAA		
NEUROD	F:CTCCTCCCCACGCGTATTCT	108	62
	R:TGATGGACAAAGGTGGGGAC		
RAX	F:CCCTGCTTTCCTTTTCCAGAAG	204	61
	R:GTGTGTAACTGGCGGGAAGAG		
OTX2	F:AATGGATTGCGGGTCCTACC	180	61
	R:TCCAGGCAGTCAGTTGTGCT		
Glast	F: TTCTCCATGTGCTTCGGCTT	142	60
	R: AGAAGAGGATGCCCAGAGGT		
Rlbp1	F: CACTATCGAGGCCGGTTACC	142	60
	R: CTCCAGAATGAAACAATATGCCTG		
Rgs9	F:GCGTGACCAATCCAAACGAA	122	60
	R: GATTCCTCCAAGGGACACCG		
Rtbdn	F: GCATGGAGCTCTGCCAGATT	135	60
	R: TGGCGTTGGCAAAAGTCTGA		
Map2	F:TGTACCTGGAGGTGGTAACGTGAA	113	60
	R:ACCTGCTTGGCGACTGTGTG		
Gapdh	F:AGGTCGGTGTGAACGGATTTG	123	60
	R:TGTAGACCATGTAGTTGAGGTCA		

### Immunocytochemistry

Cells were cultured on collagen-coated culture slides (Corning Inc., Corning, NY) and fixed in 4% paraformaldehyde at 20 °C for 10 min. The fixed cells were permeabilized with 0.1% Triton-X in TBS for 10 min and blocked in 1% bovine serum albumin (Sigma-Aldrich Corp) and 3% normal goat serum (Abcam, Cambridge, UK) in TBST for 1 h. Primary antibodies or isotype controls were incubated overnight at 4°C. The culture slides were incubated with secondary antibodies for 1 h at room temperature and were mounted with DAPI Fluoromount-G (Southern Biotechnology Associates Inc., Birmingham, AL). Images were obtained using an Axiovert200M (Carl Zeiss, Baden-Württemberg, Germany). The antibodies and isotype controls used are provided in Table 2.

**Table 2.** Antibodies and isotype control for the immunocytochemistry in this study

Antibody/isotype control	Species	Dilution	Manufacturer
RGS9	Mouse	1:50	Santa Cruz Biotechnology (sc-377252)
GLUL	Rabbit	1:500	abcam (ab73593)
Alexa Fluor™ 568	Mouse	1:2000	Invitrogen (A21043)
Alexa Fluor™ 594	Rabbit	1:500	Invitrogen (A11037)
IgM isotype control	Mouse	1:250	Invitrogen (MA1-10438)
IgG isotype control	Rabbit	1:5000	Invitrogen (02–6102)

### Western blotting

Cells were seeded at a density of 3 × 10^6^ cells in 60-mm dish and cultured overnight. Cell lysates were prepared using RIPA buffer (Thermo Fisher Scientific), Protease and Phosphatase Inhibitor Single-Use Cocktail, EDTA-free (100 ×; Thermo Fisher Scientific), and 0.5 M EDTA solution (Thermo Fisher Scientific). Protein concentration was measured using a BCA assay kit (Thermo Fisher Scientific). Next, 35 µg protein per sample was loaded into a 4–15% mini-protean precast gel (Bio-Rad Laboratories) and transferred to a membrane (Bio-Rad Laboratories). The membrane was blocked with Block Ace (KAC Co., Ltd., Kyoto, Japan) and incubated with primary antibodies overnight. After washing with TBST, the membrane was incubated with an alkaline phosphatase-conjugated secondary antibody. Chemiluminescence detection was performed using CDP-star (GE Healthcare, Chicago, IL). Band density was measured using ImageQuant (GE Healthcare). The antibodies used are provided in Table 3.

**Table 3.** Antibodies for the Western blotting in this study

Antibody	Species	Dilution	Manufacturer
ACTB	Mouse	1:1000	Santa Cruz Biotechnology (sc-69879)
GFP	Mouse	1:1000	MBL (M048-3)

### Electrophysiology

We recorded electrical responses of cells using whole-cell patch clamp techniques (Wohlschlegel *et al*. 2025). Recordings were performed in Tyrode’s solution containing 138 mM NaCl, 3 mM KCl, 1 mM CaCl_2_, 2 mM MgCl_2_, 4 mM NaOH, and 10 mM HEPES, adjusted to pH 7.4 with HCl. Patch pipettes were filled with an internal solution containing 123 mM K-gluconate, 10 mM KCl, 10 mM HEPES, 1 mM MgCl_2_, 1 mM CaCl_2_, 2 mM EGTA, 4 mM Mg-ATP, and 0.5 mM Na-GTP. Whole-cell patch pipettes had resistances of 10–15 MΩ. Electrical recordings were performed in the current-step mode. Current injections were applied for 400 ms, with stepwise decreases from 250 to − 100 pA in increments of 50 pA, and the resulting changes in membrane potential were recorded.

### RNA-seq and data processing

Total RNA was extracted from cultured cells using the Maxwell® RSC simplyRNA Tissue Kit (Promega), and mRNAs were isolated from total RNA using the NEBNext® Poly(A) mRNA Magnetic Isolation Module (New England Biolabs, Ipswich, MA). A cDNA library was constructed using the NEBNext® Ultra™ II Directional RNA Library Prep Kit for Illumina® (New Biolabs). NovaSeq X Plus (Illumina, San Diego, CA) was used to obtain paired-end 150-bp sequences of poly-A-tailed RNA. All sequence reads were processed using Fastp, version 0.24.0 for quality filtering, adaptor trimming, and removal of low-quality bases (Chen *et al*. 2018; Chen 2023). Subsequently, the quality of filtered reads was evaluated using FastQC, version 0.12.1. These reads were aligned to the NCBI rat reference genome (GRCr8) using STAR, version.2.7.11b (Dobin *et al*. 2013), and gene-level read counts were obtained using the featureCounts program in the Subread package version.2.1.1 (Liao *et al*. 2013, 2014, 2019). RNA-seq raw sequence reads were uploaded to the SRA database of NCBI. The IDs for each sample are shown in Table S1.

### Principal component analysis (PCA) and enrichment of transcriptome profiles

PCA was performed using TCC-GUI to visualize global transcriptomic differences among the four groups (Su *et al*. 2019). The analysis was based on the top 1000 most variable genes selected from the DESeq2-normalized expression data. Normalization using DESeq2 and false discovery rate (FDR) correction based on the Benjamini–Hochberg method were performed using TCC-GUI. Differentially expressed genes (DEGs) were not extracted within TCC-GUI; instead, genes with a log two-fold change > 0.5 and a *p*-value < 0.05 were selected manually from the output CSV file. Functional enrichment analysis was performed using g: Profiler with the g:GO St multiple testing correction method by applying a significance threshold of FDR < 0.05 (Kolberg *et al*. 2023). To visualize DEGs, genes upregulated in each comparison between experimental groups (log_2_ fold change > 0.5, *p* < 0.05) were mapped onto the KEGG pathway “Synaptic vesicle cycle” (KEGG pathway ID: rno04721). Mapping was performed using the KEGG Mapper – Color Tool (Kanehisa and Sato 2020; Kanehisa *et al*. 2022).

### Target gene expression analysis and quantification of cell-type identity

To evaluate the expression dynamics of specific gene groups in more detail, raw count data were normalized using the DESeq2 R package, version 1.48.1 (Love *et al*. 2014), independently of TCC-GUI processing. Based on these normalized data, expression levels across experimental groups were visualized using dot plots for the selected target genes and heat maps for retinal cell type–specific marker genes. For heat map visualization, *Z*-scores were calculated from the expression values of each marker gene (Table S2). The relative cell identity score (RCIS) was calculated by dividing the average *Z*-score of all marker genes by the standard error of the *Z*-score within each group. Here, the average *Z*-score represents the mean expression level relative to other groups, SE refers to the variability of these *Z*-scores within the group, and the calculation is based on all the marker genes included. The RCIS values were then used to evaluate the relative similarity between each group and specific retinal cell types. Group-wise comparisons of RCIS were performed using unpaired *t*-tests, and data are presented as the mean ± SE. Dot plots were generated using GraphPad Prism 4, and heat maps were generated using Morpheus software (Broad Institute Morpheus (n.d.)).

### Statistical analysis

Statistical analyses were performed using GraphPad Prism 4 software (GraphPad, San Diego, CA). Differential expression and enrichment analyses of RNA-seq data were performed using the Benjamini–Hochberg method for FDR correction. Unpaired *t*-tests were used for comparisons between groups, and Tukey’s honestly significant difference test was used for multiple comparisons. Statistical significance was set at *p* < 0.05. Error bars in graphs represent the mean ± SE.

## Results

### Establishment of Müller cell lines expressing four transcription factor genes

**Figure 1.**
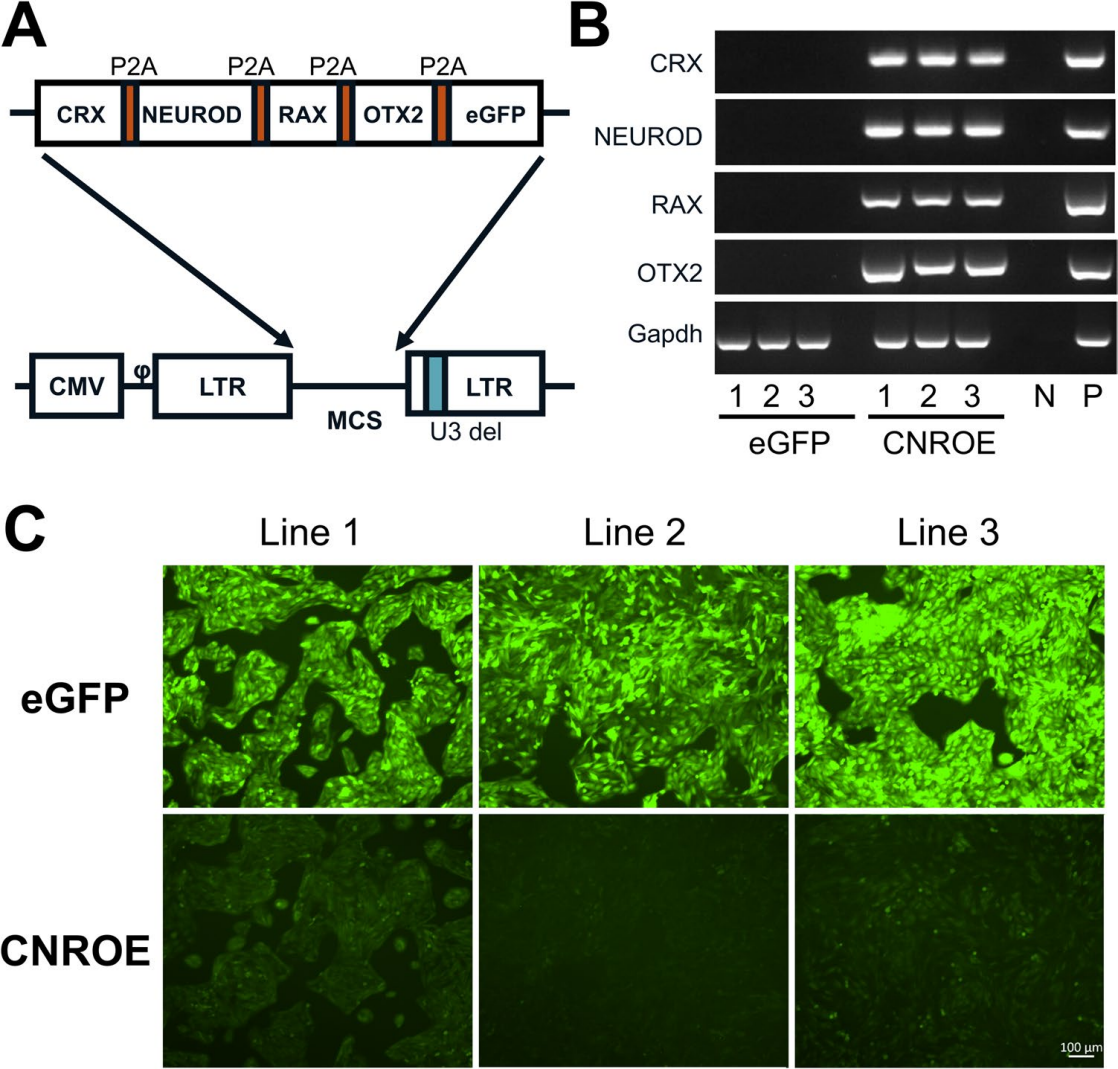
Establishment of Müller cell lines expressing four transcription factor genes. Construction of CSII-CNROE. *LTR*, long terminal repeat; *CMV*, cytomegalovirus immediate early enhancer and promoter (*A*). Agarose gel electrophoresis for detecting four transcription factor genes in each cell line. *N*:, non-template control. *P*, positive control (*B*). Representative fluorescence images of eGFP expression. *Scale bar*, 100 µm (*C*).

To evaluate whether the expression of the four transcription factors, *CRX*,* NEUROD*,* RAX*, and *OTX2*, promotes photoreceptor differentiation of rMC-1, we introduced these genes into rMC-1 cells using a polycistronic lentiviral vector (CSⅡCMV-CNROE). Subsequently, we established a stable cell line (hereafter referred to as CNROE). The construction of the expression cassette in the vector CSⅡCMV-CNROE is shown in Fig. 1*A*. We used the control lentiviral vector CSⅡCMV-eGFP, which lacks the four transcription factor genes, to generate a stable cell line (referred to below as eGFP). Exogenous transcripts of the four factors were specifically expressed in CNROE but not in eGFP, as confirmed using RT-PCR (Fig. 1*B*). In contrast, variability in EGFP fluorescence intensity was observed among CNROE clones (Fig. 1*C*).

### Quality assessment, mapping, and gene-level read quantification of RNA-seq data

Raw sequencing data quality was assessed using FastQC. Sufficient read counts and consistent average read lengths were confirmed across all samples (Table S3). No significant bias in GC content was observed, and no adapter contamination was present. Therefore, the obtained reads were judged to be of high quality and suitable for downstream analysis. Trimmed reads were aligned to the rat reference genome (GRCr8) using STAR. Generally, high mapping rates (89.7–95.3%) were observed across all samples (Table S4). Gene-level read quantification was performed using featureCounts (Table S5). Most reads (79.2–81.5%) were successfully assigned to genes. The remaining unassigned reads were primarily owing to multimapping, reads not overlapping annotated genes (No Feature), or ambiguous assignments (Ambiguous). Collectively, these results confirm that the RNA-seq data possess sufficient quality and depth to support downstream gene expression analysis.

### Effect of CNROE on neuronal and ECM-related gene expression

**Figure 2.**
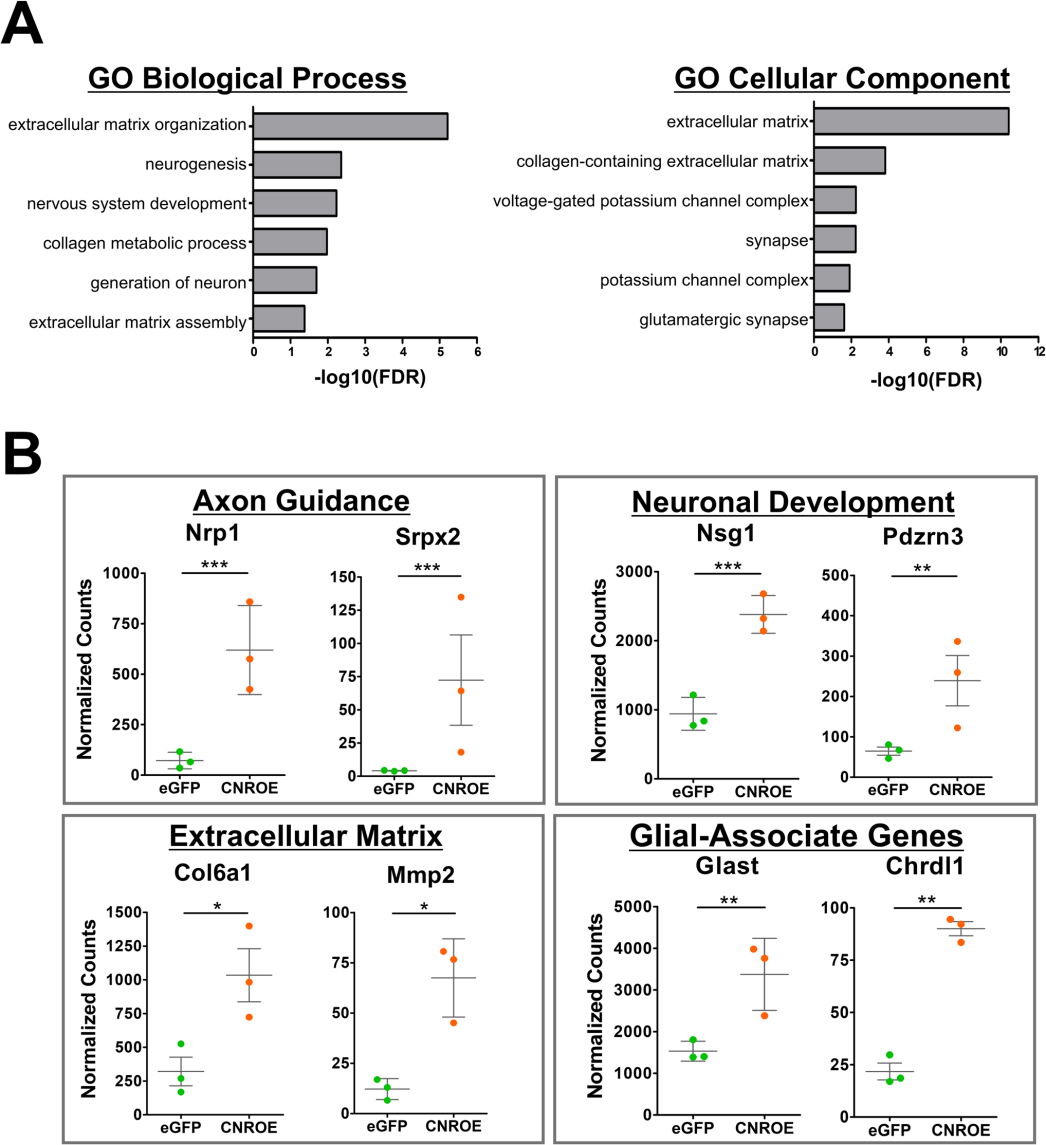
Effect of CNROE on rMC-1’s neuronal and ECM-related gene expression. *Bar graphs* show the neural- and extracellular matrix-related GO terms of genes upregulated in the CNROE compared with eGFP (*A*). Normalized counts related to axon guidance, neuronal development, ECM formation, and glia-associated genes. Statistical significance was determined using the FDR calculated by Benjamin Hochberg.*, **, *** FDR < 0.05, 0.01, 0.001. Data are presented as mean ± SE values (*B*).

Enrichment analysis was performed by identifying upregulated genes with log_2_ fold change > 0.5 and *p* < 0.05, followed by GO analysis of these genes. The GO terms in biological processes based on the upregulated gene sets were extracellular matrix organization (GO:0030198), extracellular matrix (ECM), and nervous system development, neurogenesis (GO:0022008), nervous system development (GO:0007399), collagen metabolic processes (GO:0032963), generation of neurons (GO:0048699), and extracellular matrix assembly (GO:0085029). GO terms in the cellular component category included extracellular matrix organization (GO:0030198), collagen-containing extracellular matrix (GO:0062023), voltage-gated potassium channel complex (GO:0008076), synapse (GO:0045202), potassium channel complex (GO:0034705), and glutamatergic synapse (GO:0098978) (Fig. 2*A*). Thus, CNROE transduction mainly induced gene expression related to neural differentiation and the extracellular matrix. Among the genes with increased expression, we found marked changes in *Nrp1* and *Srpx2*, which promote axon guidance, and in the neurogenesis-related genes *Nsg1* and *Pdzrn3* (*q*-value < 0.05). We also found that the expression levels of *Col6a1* and *Mmp2* were markedly increased. Interestingly, *Glast* and *Chrdl1*, which are known glial markers, were also significantly upregulated despite being functionally distinct from neurodevelopmental genes (Fig. 2*B*). The GO analysis of DEGs that were significantly upregulated at an FDR < 0.05 is presented in Fig. S1A.

### Comparisons of the relative cell identity score of retinal cells in CNROE- and eGFP-expressing cell lines

**Figure 3.**
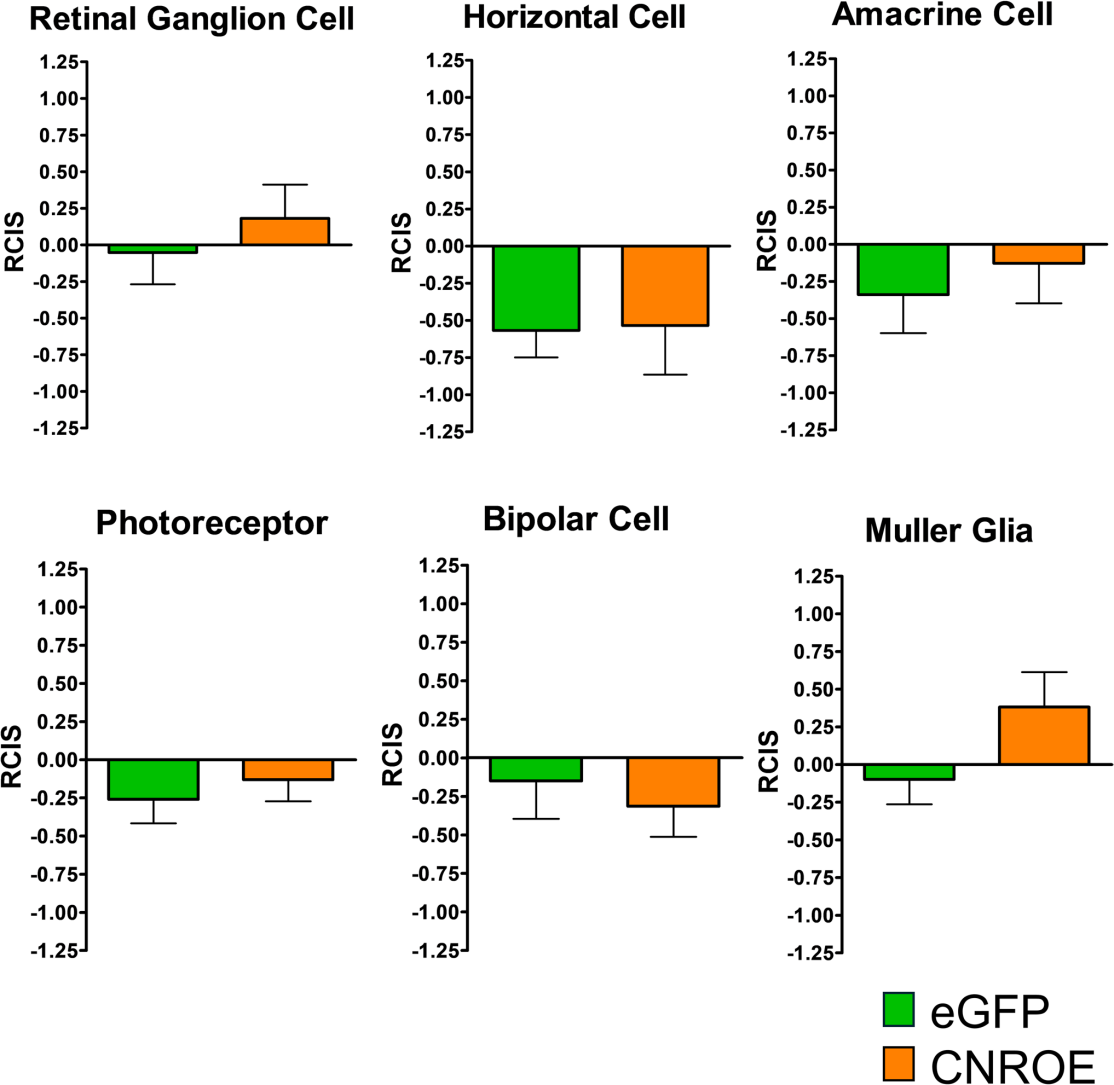
Comparison of relative cell identity score (RCIS) of retinal cells in CNROE-expressing and eGFP-expressing cell lines. Data are presented as the mean ± SE values (*n* = 3, unpaired *t*-test, *p < 0.05). No statistically significant differences were observed between the groups.

We used the RCIS to investigate the direction of differentiation in CNROE-transduced cells. The marker genes used in the RCIS to characterize each cell type are summarized in Table S2. The RCI scores of retinal ganglion cells (RGCs) and glial cells, including Müller cells among CNROE cells, were higher than those in eGFP cells (Fig. 3).

### Effects of the culture medium on the gene expression in CNROE- or eGFP-transduced rMC-1 cells

**Figure 4.**
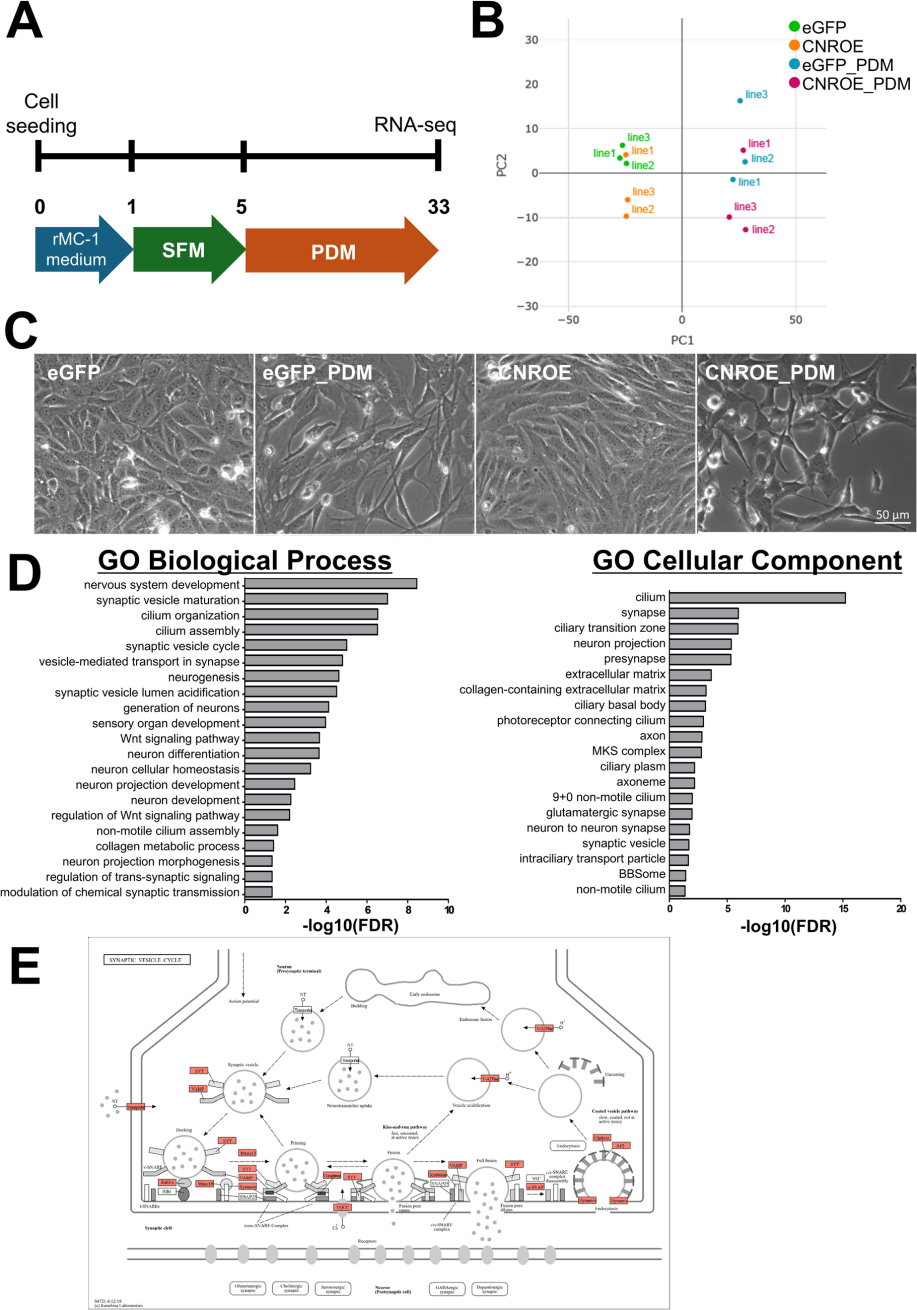
Effects of the culture medium on gene expression in CNROE and eGFP. Experimental protocol for the induction of photoreceptor-like cells. *SFM*, serum-free medium; *PDM*, photoreceptor differentiation medium (*A*). PCA plot was created using gene expression data (*B*). Morphology of cells at day 33. *Scale bar*, 50 µm (*C*). Neural and extracellular matrix-related GO terms (*D*) and KEGG pathway map of the human synaptic vesicle cycle (*E*) of genes upregulated in CNROE_PDM compared with eGFP.

To promote the differentiation of rMC-1 cells toward neuronal cells, the culture medium was replaced with a medium containing valproic acid, CHIR-99021, forskolin, retinoic acid, and DAPT, which can promote differentiation into photoreceptor cells. Based on previous studies, we selected compounds known to enhance photoreceptor differentiation and cultured eGFP and CNROE in PDM (Fig. 4*A*). To evaluate the overall transcriptomic differences among the four experimental groups, eGFP, CNROE, eGFP_PDM, and CNROE_PDM, PCA was performed. The PCA plot revealed a clear separation, primarily driven by the compound treatment, with CNROE and CNROE_PDM distinctly separated from eGFP and eGFP_PDM (Fig. 4*B*). This indicates that compound treatment exerts a stronger influence on gene expression profiles than that of gene introduction. To examine cellular morphology in greater detail, cells were reseeded at low density on day 33 and subsequently analyzed. eGFP cultured in PDM (eGFP_PDM), and CNROE cultured in PDM (CNROE_PDM) exhibited elongated processes compared with eGFP and CNROE (Fig. 4*C*).

Next, to investigate the signaling pathways and cellular components upregulated in the eGFP and CNROE_PDM groups, we performed Gene Ontology (GO) enrichment analysis. As in the previous GO analysis, we applied an exploratory threshold of log_2_ fold change > 0.5 and *p*-value < 0.05, considering the limited number of DEGs identified using the more stringent cutoff (*q*-value < 0.05). The analysis showed that terms, including nervous system development (GO:0007399) and extracellular matrix (GO:0031012), which were previously identified as enriched in CNROE, were significantly enriched. Additionally, some cilia-related terms, including cilium organization (GO:0044782) and cilium (GO:0005929); terms related to synaptic vesicle formation and secretion, such as synaptic vesicle cycle (GO:0099504) and synapse (GO:0045202); eye development–associated terms like sensory organ development (GO:0007423); and terms involved in photoreceptors morphogenesis such as photoreceptor-connecting cilium (GO:0032391) were also enriched in the upregulated gene set (Fig. 4*D*). Based on the enrichment of synaptic vesicle-related GO terms, we used KEGG Mapper to map the upregulated genes to the synaptic vesicle cycle pathway. In all, 34 genes were mapped to the pathway (Fig. 4*E*). The GO analysis of DEGs significantly upregulated at an FDR < 0.05 is presented in Fig. S1B.

### Expression changes in Müller glia, photoreceptor transcription factors and specific genes, and neural retina genes induced by CNROE and PDM

**Figure 5.**
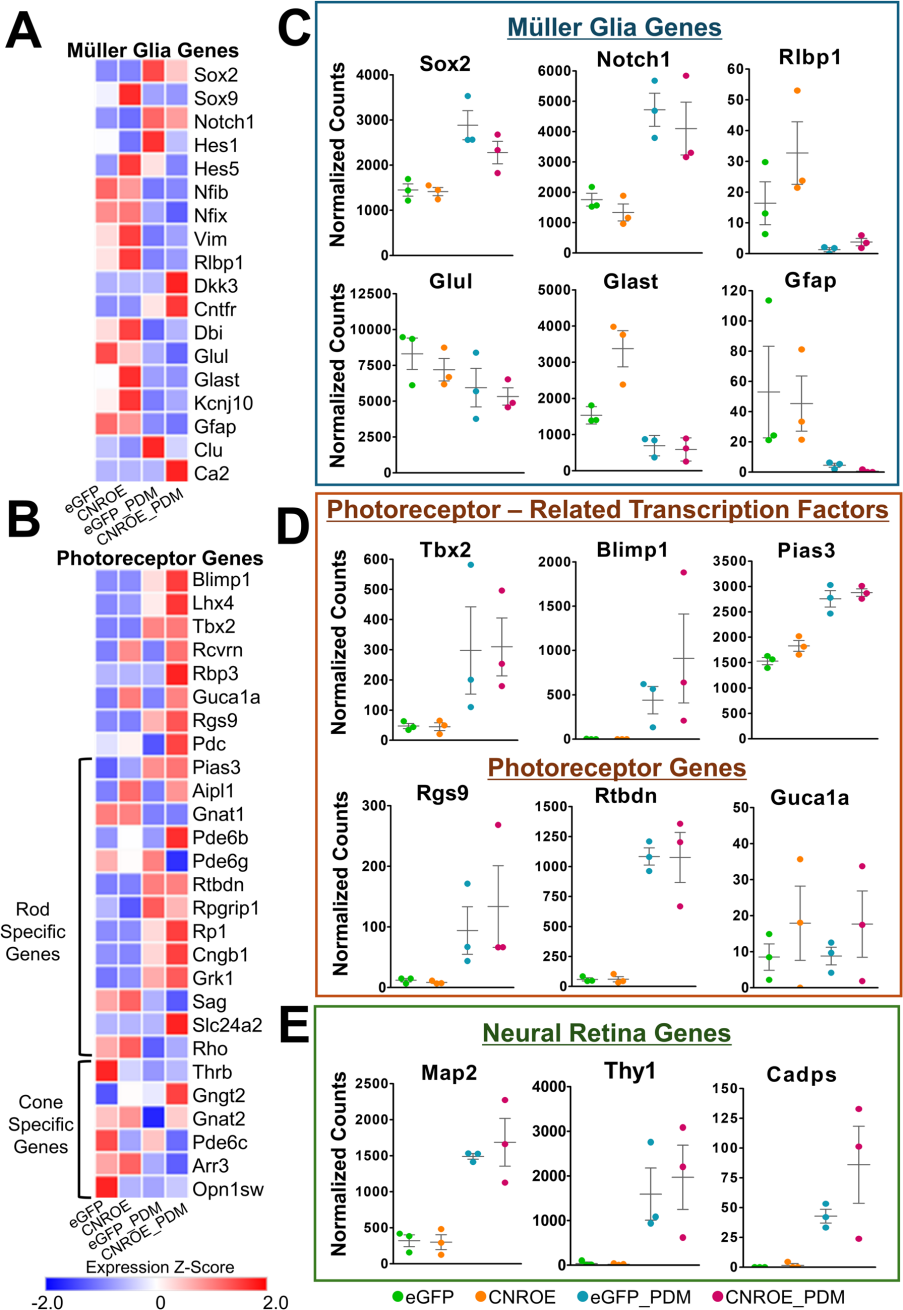
Expression changes in Müller glia, photoreceptor, and neural retina–related genes induced by CNROE and PDM. Heat maps showing the expression of Müller glial transcription factors and specific genes (*A*) and Müller cell–specific genes (*B*). Expression heat maps of photoreceptor transcription factors and specific genes (*C*), photoreceptor-related transcription factors and specific genes (*D*), and neural retina genes (*E*). Data are presented as the mean ± SE values (*n* = 3).

In the group cultured in PDM, several photoreceptor-related genes showed a tendency toward increased expression, whereas Müller glia–related genes tended to be downregulated (Fig. 5*A*, B). In this group, genes involved in maintaining Müller cell function, such as *Glast*,* Rlbp1*,* Glul*, and *Gfap*, showed a downward trend, whereas the dedifferentiation-related transcription factors *Sox2* and *Notch1* exhibited an upward trend in expression (Fig. 5*C*). Cells cultured under PDM conditions showed increased expression of photoreceptor-related transcription factors, including *Tbx2*,* Blimp1*, and *Pias3*, as well as genes involved in photoreceptor light response and metabolic regulation, such as Rtbdn and Rgs9. In contrast, Guca1a exhibited an increased expression trend in CNROE and CNROE_PDM (Fig. 5*D*). The expression of the neuron-specific genes *Map2*,* Thy1*, and *Cadps* was also increased in the PDM culture group (Fig. 5*E*).

Among genes showing expression changes in the transcriptome analysis, Müller glial markers (*Glast* and *Rlbp1*), photoreceptor markers (*Rtbdn* and *Rgs9*), and a neuronal marker (*Map2*) were selected, and their temporal expression changes at 2, 3, and 4 wk after differentiation induction were analyzed by RT-qPCR. Expression levels of the Müller glial marker Glast were consistently lower in eGFP_PDM and CNROE_PDM than in both eGFP and CNROE from week 2 onward. *Rlbp1* also showed a significant decrease at week 4 in the eGFP_PDM and CNROE_PDM conditions relative to CNROE (*p* < 0.05) (Fig. S2*A*). Among photoreceptor markers, *Rtbdn* expression did not show marked changes across any conditions or time points. In contrast, *Rgs9* exhibited condition- and time-dependent expression dynamics. At week 3 (day 26), CNROE induced a significant increase in *Rgs9* expression compared with both eGFP and CNROE (*p* < 0.001), whereas CNROE_PDM showed a significant decrease in *Rgs9* expression relative to eGFP_PDM (*p* < 0.05). At week 4, although no statistically significant differences were observed, *Rgs9* expression in the CNROE_PDM condition tended to surpass that in the eGFP_PDM condition (Fig. S2*B*). The neuronal marker *Map2* was consistently upregulated in the eGFP_PDM and CNROE_PDM conditions from week 2 onward compared with both eGFP and CNROE conditions (*p* < 0.01) (Fig. S2*C*).

### Clonal differences in transcription factor expression and in Müller glia, photoreceptor, and neural retina gene expression

In the polycistronic expression cassette of CSⅡ-CMV-CNROE, the fluorescent reporter eGFP was positioned downstream of the four transcription factors, enabling eGFP protein expression to serve as a proxy for the overall expression level of transcription factors derived from the same cassette. Western blot analysis revealed clear differences in eGFP protein levels among clones. Notably, clone 2 exhibited approximately one-third of the eGFP expression observed in clones 1 and 3 (Fig. S3*A*, *B*).

To examine how differences in transcription factor expression relate to cellular state, we performed RNA-seq–based transcriptome analysis and compared the expression of differentiation-related genes among clones. We analyzed the induction of neuronal-associated genes and suppression of donor Müller glia–associated gene expression during differentiation. Neuronal-associated genes *Syt1*,* Map2*, and *Guca1a* were consistently expressed at lower levels in clone 2 than in clones 1 and 3 (Fig. S3*C*). In particular, *Syt1* and *Map2* showed pronounced differences between clone 2 and the other clones, and their reduced expression persisted even after culture in PDM. Although *Guca1a* exhibited an increasing trend in CNROE groups, its expression remained lower in clone 2 than in the other clones. We also examined Müller glia–associated genes *Rlbp1*,* Glul*,* Nfix*, and *Vim* (Fig. S3*D*). Although the differences in *Rlbp1*,* Glul*, and *Nfix* expression among clones were reduced after culture in PDM, *Vim* expression remained elevated in clone 2 even under differentiation conditions.

### Effects of CNROE and PDM on protein expression and electrophysiological function

Immunocytochemical analyses revealed that the signal intensity of GLUL, a Müller glia marker, was strongly positive in cells cultured in rMC-1 medium. By comparison, in cells cultured in differentiation medium, the strong GLUL signal observed before differentiation was markedly attenuated, decreasing to a weak fluorescence level comparable to that of the negative control. Meanwhile, expression of the photoreceptor marker RGS9 exhibited a diffuse distribution throughout the entire cell both before and after differentiation; however, a slight increase in signal intensity was observed following differentiation induction (Fig. S4).

Electrophysiological analyses revealed that cells cultured in PDM exhibited a subset of cells that displayed rebound burst firing following current injection (Fig. S5*A*). Rebound burst firing was frequently observed in eGFP_PDM, whereas it was less frequently detected in CNROE_PDM, with both the frequency and amplitude of firing reduced. No burst rebound firing was observed in conditions eGFP and CNROE. Transcriptomic analysis of molecules associated with rebound burst firing revealed that, compared to eGFP, cells in conditions eGFP_PDM and CNROE_PDM showed increased expression of *Cacna1g*, encoding the T-type calcium channel Cav3.1, and *Kcnn2*, encoding the calcium-activated potassium channel SK2 (Fig. S5*B*).

### CNROE_PDM exhibits gene expression profiles characteristic of photoreceptors

**Figure 6.**
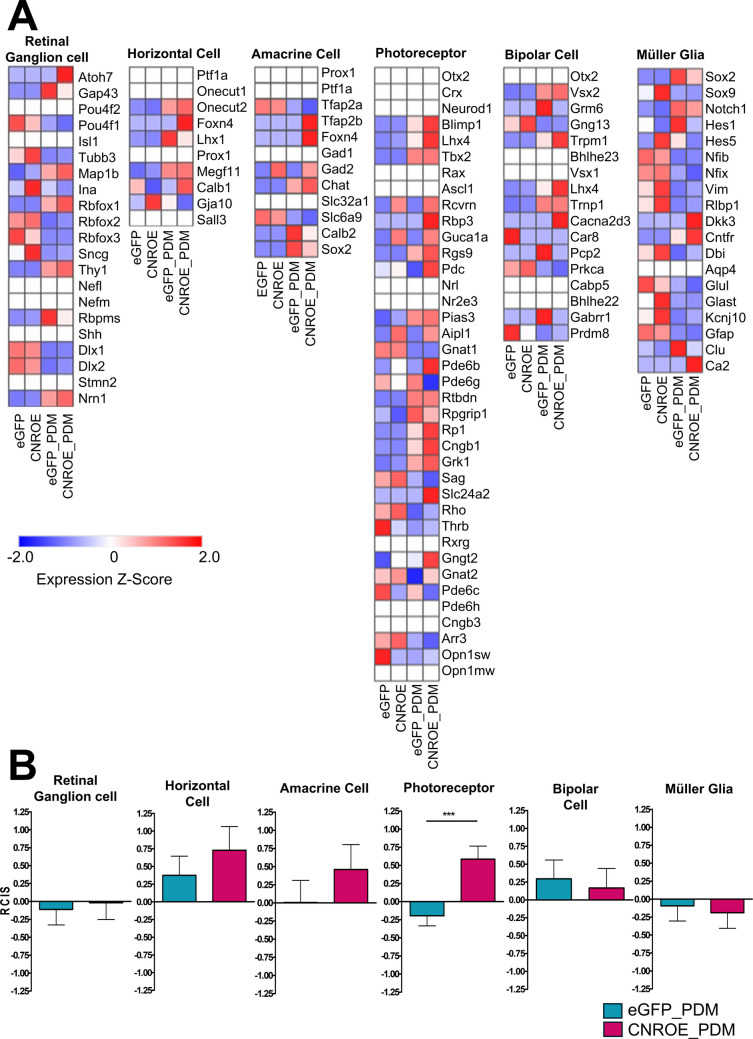
CNROE_PDM exhibits gene expression profiles characteristic of photoreceptors. Heat maps of the expression of retinal neurons and Müller glial transcription factors and the specific genes (*A*). Relative cell identity scores (RCIS) of retinal neurons and Müller glia in eGFP_PDM and CNROE_PDM (*B*). Data are presented as mean ± SE values (*n* = 3, unpaired *t*-test, ***p < 0.001).

To quantitatively assess the extent of differentiation in eGFP_PDM and CNROE_PDM, we generated a heat map of retinal cell-marker gene expression and calculated the RCIS. The RCIS was also calculated for each retinal neuronal cell type. In both eGFP_PDM and CNROE_PDM, a downward trend was observed in the RCIS for RGCs and Müller glia, which showed relatively high CNROE scores. Furthermore, the RCIS for photoreceptors was significantly higher in the CNROE_PDM condition than in the eGFP_PDM condition (Fig. 6).

The stability of the RCIS may be influenced by the size of the marker gene set. In particular, for horizontal cells, amacrine cells, and bipolar cells, the number of marker genes is relatively small owing to the selection of genes intended to cover the entire developmental trajectory from early progenitors to mature cell functions. To assess this aspect, we performed a sensitivity analysis by varying the number of marker genes. Specifically, for each cell type, 30%, 50%, or 70% of genes from the original marker list were randomly sampled, and RCIS was recalculated under each condition. We observed that, for all sampling percentages, the relative ordering of RCIS across the four groups was consistent with that obtained using the full set of marker genes (100%). In contrast, when only 30% of genes were sampled, the variance of RCIS was relatively high. Variance decreased at 50–70% sampling, indicating greater stability of RCIS under these conditions (Fig. S6, Table. S6). These results indicate that, while RCIS can be affected by very small marker gene sets, a sufficiently large number of genes allows RCIS to provide a stable and robust measure for relative comparisons between cell types and experimental conditions.

## Discussion

In this study, to explore a potential treatment method for retinal dystrophy using direct reprogramming, we attempted to induce the differentiation of photoreceptors in retinal Müller cells by introducing four transcription factors (*CRX*,* RAX*,* NEUROD*, and *OTX2*) using a polycistronic vector and by applying small-molecule compounds.

In CNROE, compared to eGFP, the significant upregulation of *Nrp1* and *Srpx2*, which promote axon guidance, as well as Nsg1 and *Pdzrn3*, which are involved in neurogenesis, suggests that the introduction of these four factors promotes differentiation toward a neuronal lineage (Erskine *et al*. 2017; Baizabal *et al*. 2018; Cong *et al*. 2020; Qi *et al*. 2023). Additionally, Col6a1 and *Mmp2*, which are induced during extracellular matrix remodeling associated with axonal extension, were upregulated (Chan *et al*. 2020). CNROE exhibited a higher RCIS for RGCs compared to that of eGFP, along with elevated expression of *Tubb3*,* Ina*,* Sncg*, and *Syt13*, which are characteristic markers of mid-developmental stage RGCs. These findings collectively suggest that CNROE exhibit features resembling those of early-to mid-stage RGCs that have begun axonal outgrowth (Sharma and Netland 2007; Mu *et al*. 2008; Surgucheva *et al*. 2008). However, CNROE also exhibited significant upregulation of some Müller glia–specific genes, along with a tendency for increased RCIS, suggesting that these cells also possess the features of this lineage. The differentiation of CNROE toward the RGC lineage is thought to be influenced by NEUROD. NEUROD not only facilitates differentiation toward photoreceptors but also acts as a master control gene that independently induces differentiation into early-born retinal neurons, such as RGCs, Horizontal cells, and Amacrine cells (Le *et al*. 2006). These findings suggest that NEUROD may have directed the cells toward the RGC fate, resulting in enhanced expression of RGC-specific features. The upregulation of certain Müller glia–related genes in CNROE may be explained by a cellular response in which four transcription factors promote a shift toward neuronal differentiation but fail to fully commit the cells to a neuronal fate. This unstable intermediate state may have triggered a compensatory tendency for the cells to revert to the Müller glial identity. During reprogramming, cells can enter a metastable state in which genes associated with the original cell identity are reactivated as part of a homeostatic response. This phenomenon has also been observed in studies on induced pluripotent stem cells in which donor cell-type lineage-specific genes are reactivated in cells that do not acquire pluripotency through the introduction of Yamanaka factors (Tanabe *et al*. 2013; Gill *et al*. 2022; Singh and Zhakupova 2022). Based on the above results and previous findings, it is suggested that, although the four transcription factors possess the ability to promote neuronal differentiation, they are insufficient to drive full neuronal maturation on their own. Instead, the cells appear to be in a dynamic equilibrium between an immature neuronal state of RGCs and a reversion toward the original identity of Müller glia, possibly because of opposing forces. This observation is consistent with earlier studies reporting that cells transduced with four transcription factors remained in an early stage of differentiation, albeit targeting a different donor cell type (Fukuda *et al*. 2018). The relatively low expression level of each gene may have resulted from the use of a polycistronic expression system, which involved a longer transgene cassette than that of monocistronic systems. Additionally, the establishment of stable cell lines from genetically transduced cells may have further contributed to the reduced expression. Consistent with this interpretation, our analyses indicate that lower overall expression levels of transcription factors derived from the polycistronic cassette are associated with an altered transcriptional state. Specifically, conditions characterized by reduced transgene expression showed the insufficient upregulation of neuronal maturation-related genes together with the persistent expression of Müller glia–associated markers. These findings support the findings of previous reports suggesting that transcription factor dosage plays an important role in determining reprogramming efficiency and the extent of neuronal maturation. However, the present analyses were based on RNA-seq data and reporter protein expression as indirect indicators of transcription factor levels and thus did not include direct quantification of individual transcription factor proteins. Future studies incorporating protein-level analyses of each transcription factor will be required to define the relationship between transcription factor dosage and neuronal maturation more precisely.

To promote differentiation into photoreceptors, differentiation was induced using PDM, which contained five compounds previously utilized for the pharmacological reprogramming of fibroblasts to photoreceptors (Mahato *et al*. 2020). CNROE cultured in PDM showed changes in the expression of genes associated with synaptic vesicle formation, cilia structure, and sensory organ development compared to those in eGFP. These findings suggest that the cells are at the developmental stage of neuronal morphology and function. Enrichment of these GO terms was also observed in eGFP_PDM. Principal component analysis revealed a clear separation between cells cultured in rMC-1 medium and those cultured in PDM, whereas the boundary between eGFP_PDM and CNROE_PDM was less distinct, suggesting that the two groups shared relatively similar characteristics. These findings indicate that in the current experimental system, PDM plays a significant role in promoting the acquisition and maturation of neuronal functions. In contrast, the number of genes enriched in the photoreceptor-connecting cilium (GO:0032391), which is essential for photoreceptor morphogenesis, was higher in CNROE_PDM than in eGFP_PDM.

To further interpret the biological significance of transcriptomic changes observed in CNROE_PDM compared with those in eGFP, we performed KEGG pathway analysis on the upregulated DEGs. This analysis identified significant enrichment of the synaptic vesicle cycle pathway (rno04721), with a total of 34 genes mapped to this pathway, which comprises key components involved in synaptic vesicle docking, fusion, and recycling in neurons. Although the enrichment of synaptic vesicle–related genes can sometimes reflect changes in general secretory or membrane trafficking pathways, a closer inspection of the mapped genes revealed the presence of multiple molecules that are well established as core regulators of neuronal synaptic vesicle release and recycling. These include synaptotagmin-1 (*Syt1*), vesicle-associated membrane protein 2 (*Vamp2*), syntaxin-1A/1B (*Stx1a/1b*), syntaxin-binding protein 1 (*Stxbp1*), *Rab3a*, Unc13a (*Munc13-1*), and dynamin-1 (*Dnm1*), which collectively constitute essential components of neuron-specific vesicle control machinery rather than generic secretion systems. Functionally, *Syt1* acts as a principal Ca^2+^ sensor for fast neurotransmitter release (Grassmeyer *et al*. 2019), while *Vamp2* and *Stx1a/1b* form the central elements of the SNARE complex required for synaptic vesicle fusion (Jahn *et al*. 2024). Furthermore, *Unc13a (Munc13-1)* is indispensable for synaptic vesicle priming and acquisition of release competence (Dittman 2019). Notably, several of these molecules have been implicated in synaptic function within retinal neurons, including photoreceptors: *Stxbp1* regulates vesicle release at photoreceptor ribbon synapses (Kakakhel *et al*. 2020), *Rab3a* participates in vesicle trafficking at photoreceptor synapses (Tian *et al*. 2012), and *Dnm1* is required for synaptic vesicle recycling in retinal neurons (Hanke-Gogokhia *et al*. 2024). Collectively, these findings suggest that enrichment of the synaptic vesicle cycle pathway reflects the partial activation of a neuron-specific synapse-related transcriptional program rather than a mere increase in generic secretory activity. However, because synapse formation and neurotransmission were not directly assessed, these changes should not be interpreted as evidence of fully mature or functional synapses.

Our results indicate that cells cultured in PDM exhibit substantial heterogeneity in their electrophysiological properties, suggesting the coexistence of immature cells and cells in which electrical responsiveness is partially established. Rebound burst firing was relatively frequent in eGFP_PDM, whereas in CNROE_PDM, both the frequency and amplitude of rebound burst firing following stimulus offset were reduced. Rebound burst firing observed after stimulus offset results from de-inactivation of Cav3.1 channels encoded by Cacna1g, leading to Ca^2+^ influx, and is a hallmark electrophysiological feature of immature neurons (Chen *et al*. 2005). In CNROE_PDM, *Kcnn2* expression was stably detected. SK2 channels are known to suppress both the frequency and amplitude of spike bursts (Ohtsuki and Hansel 2018) and are expressed in rodent retinal neurons from postnatal day 1, contributing to the regulation of membrane potential responses (Klöcker *et al*. 2001). In CNROE_PDM, Cacna1g expression was slightly lower than in eGFP_PDM, consistent with the observed reduction in rebound burst firing frequency and amplitude. Notably, *Cacna1g* expression decreases as neuronal differentiation progresses (Bertolesi *et al*. 2003). Together, these observations suggest that cells in CNROE_PDM exhibit a greater shift toward a neuronal fate and have partially acquired mechanisms for controlling rebound burst firing compared to eGFP_PDM.

Immunofluorescence analysis revealed that cells cultured under differentiation conditions exhibited a clear reduction in the signal intensity of GLUL, a Müller glia marker, whereas expression of the photoreceptor marker RGS9 was modestly increased. These findings suggest that, at the protein level, the cells are gradually losing their Müller glia identity but have not yet undergone full conversion into mature photoreceptors, indicating that they may reside in a transitional state. Previous studies using early photoreceptor progenitors derived from human iPS cells have reported that photoreceptor-associated markers such as Recoverin and CRX fail to display the polarized subcellular localization characteristic of mature photoreceptors and instead show a diffuse intracellular distribution (Wiley *et al*. 2016). This pattern is consistent with the distribution of RGS9 observed in the present study and supports the notion that diffuse expression of photoreceptor markers is a common feature of immature photoreceptor progenitor states. Notably, RGS9 signals in the present study were uniformly detected across the entire cell population without enrichment in specific subcellular regions or subsets of cells. This observation suggests that differentiation or reprogramming did not preferentially occur in a limited fraction of cells but rather progressed relatively synchronously across the population, leading to a global shift toward an early photoreceptor-like state.

Moreover, CNROE_PDM exhibited a significantly higher RCIS for photoreceptors than that of eGFP_PDM, suggesting that the four transcription factors contribute to photoreceptor-directed differentiation. Taken together, these findings suggest that the addition of compounds may have unmasked the latent effects of *CRX* in CNROE, thereby overriding both the early-born neuron-directed differentiation induced by NEUROD and the competing tendency to revert toward a Müller glia–like fate, ultimately promoting differentiation toward a photoreceptor identity.

Our results suggest that the combination of transcription factor introduction and PDM promotes a shift toward a photoreceptor-like fate. However, the absence of the upregulation of phototransduction-related genes, such as *Rho*,* Opn1sw*, and *Opn1mw*, as well as the lack of reporter gene activation, indicates that the cells remain in an immature photoreceptor-like state. This immature state is likely maintained by the increased expression of *Sox2* and *Notch1* observed under these conditions. *Sox2* and Notch1 are known to promote cellular dedifferentiation and maintain an undifferentiated state while inhibiting differentiation (Graham *et al*. 2003; Kageyama *et al*. 2008; Del Debbio *et al*. 2016; Gorsuch *et al*. 2017). Notably, *Notch1* suppresses photoreceptor differentiation by inhibiting key transcription factors required for photoreceptor development, including *Crx*,* Otx2*, Neurod1, and *Nrl* (Jadhav *et al*. 2006). In addition, valproic acid (VPA, an HDAC inhibitor) and CHIR-99021 (a GSK3β inhibitor) included in PDM promote *Sox2* and *Notch1* activation (Lyssiotis *et al*. 2007; Adler *et al*. 2008; Esfandiari *et al*. 2012; Bang *et al*. 2013; Zheng and Conner 2018; Delepine *et al*. 2021). VPA has been shown to induce *SOX2* expression and maintain an undifferentiated state in neural stem and progenitor cells (Bang *et al*. 2013). Similarly, CHIR-99021-mediated GSK3β inhibition preserves the undifferentiated state in neural progenitors and brain organoid models by sustaining Notch-related gene activity (Delepine *et al*. 2021). These findings support our observation that elevated *Sox2* and *Notch1* levels contribute to the maintenance of an immature photoreceptor-like state in our system. Together, these observations highlight that adjusting the timing or dosage of VPA and CHIR-99021 may be critical for promoting mature photoreceptor differentiation (Esfandiari *et al*. 2012; Delepine *et al*. 2021). Demonstrating this experimentally remains a future challenge. Our current results provide a mechanistic rationale linking *Sox2*/*Notch1* activation to the maintenance of an immature photoreceptor-like state, suggesting that future optimization of these conditions could enhance the efficiency of photoreceptor maturation.

In this study, we used rMC-1, an immortalized Müller glial cell line generated by SV40 large T antigen, and demonstrated that cell fate can be shifted toward a neuronal lineage through the introduction of transcription factors combined with treatment with small molecules. Although the induction of fully mature photoreceptor markers was not achieved, RNA-seq analysis revealed the partial activation of neuronal- and photoreceptor-related genes, suggesting that immortalized cell lines retain a certain degree of reprogramming capacity. SV40-mediated immortalization is known to cause constitutive activation of the cell cycle through inhibition of the p53 and Rb pathways, thereby affecting cell-cycle regulation and epigenetic states (Sáenz Robles *et al*. 2013). Such conditions may be unfavorable for neuronal differentiation and reprogramming, which require cell-cycle exit and stabilization of chromatin states. Indeed, previous studies have shown that the transition to the G0/G1 phase and cell-cycle arrest are critical for the acquisition of stable neuronal identities during neuronal differentiation and direct reprogramming (Valls 2003). Moreover, the induction of neuronal gene expression requires extensive epigenetic reorganization, including histone modifications and chromatin accessibility remodeling; the disruption of these processes has been suggested to limit the degree of differentiation and maturation (Polo *et al*. 2010). In addition to its effects on the p53/Rb pathways, SV40 large T antigen has been reported to interact with epigenetic regulators such as CBP/p300 and HDAC1, potentially altering histone modification states and transcriptional regulation (Hardwick and Philpott 2014). Considering that immortalization itself may reshape the epigenetic landscape, differentiation control in immortalized cell lines is likely to be more constrained than in primary Müller glia or in vivo systems. Furthermore, the p53 pathway has been shown to contribute to neuronal reprogramming through *SOX2* (Wang *et al*. 2016), highlighting the close interplay between cell-cycle regulation, epigenetic states, and neuronal fate determination. Overall, SV40-immortalized Müller glia should be regarded as a model with inherent limitations for neuronal differentiation. Despite these constraints, the modulation of culture conditions in this study—specifically, suppression of proliferation by serum withdrawal combined with signaling control using small molecules—induced detectable changes in cellular state. Under differentiation conditions, RNA-seq analysis showed upregulation of *Sox2* and *Notch1*, whereas RT-qPCR analysis revealed significant downregulation of the Müller glia–specific genes *Glast* and *Rlbp1*, suggesting the attenuation of glial identity. In addition, significant upregulation of the neuronal marker *Map2* and the photoreceptor-related gene *Rgs9* was observed, indicating a partial transition toward a transcriptional state closer to neuronal lineages, potentially via an undifferentiated or neural progenitor-like state. Overall, the present results do not indicate that SV40-immortalized Müller glia possess differentiation potential comparable to that of primary Müller glia or in vivo Müller glia. However, our findings demonstrate that, even under the constraints imposed by immortalization, the manipulation of proliferative status and intracellular signaling can induce transcriptomic reorganization reflecting changes in cellular state, leading to a partial shift toward neural progenitor-like or neuronal transcriptional profiles.

Future studies will need to optimize combinations and expression levels of reprogramming factors and introduce additional maturation-promoting cues to determine the extent to which transcriptomic changes can be coupled to functional neuronal maturation. In addition, because the present analyses were primarily based on bulk transcriptomic data and indirect immunocytochemical observations, future studies incorporating single-cell-level approaches, such as single-cell RNA sequencing and quantitative imaging analyses, will be important to resolve cellular heterogeneity and to more precisely define transitional cell states during reprogramming. Importantly, such approaches would further enhance the utility of this system as a platform for dissecting the mechanisms underlying Müller glial plasticity and neuronal fate acquisition at higher resolution.

## Conclusion

The screening cell line established in this study is expected to serve as a valuable tool for advancing the development of retinal disease models and facilitating drug discovery efforts. Compared to primary cultured cells, immortalized cell lines have the significant advantage of unlimited proliferative capacity, making them well suited for establishing screening platforms. Furthermore, reports demonstrating even partial neuronal or photoreceptor reprogramming in immortalized cell lines are limited, highlighting the novelty of our findings. Overall, these results suggest potential applications of immortalized Müller glial cell lines in future retinal regeneration research, disease model development, and drug screening.

## Supplementary information

Below is the link to the electronic supplementary material.

ESM 1 (DOCX 4.80 MB)

## Data Availability

The RNA-seq datasets generated during the current study are available in the NCBI Sequence Read Archive (SRA) under BioProject ID PRJNA1295714. A detailed sample and run information are provided in Supplementary Table S1. Other datasets used and/or analyzed during the current study are available from the corresponding author upon reasonable request.
