# Supplementary material for: Direct reprogramming of Müller glia into photoreceptors via multiple transcription factors and small molecules: molecular mechanisms and transcriptomic analysis
Source: In Vitro Cell Dev Biol Anim. 2026 Feb 25;62(5):685–703. doi: 10.1007/s11626-026-01164-0 (PMC13246565; doi:10.1007/s11626-026-01164-0)
Supplement: Supplementary file 1 — (DOCX 4.80 MB) [file 11626_2026_1164_MOESM1_ESM.docx]

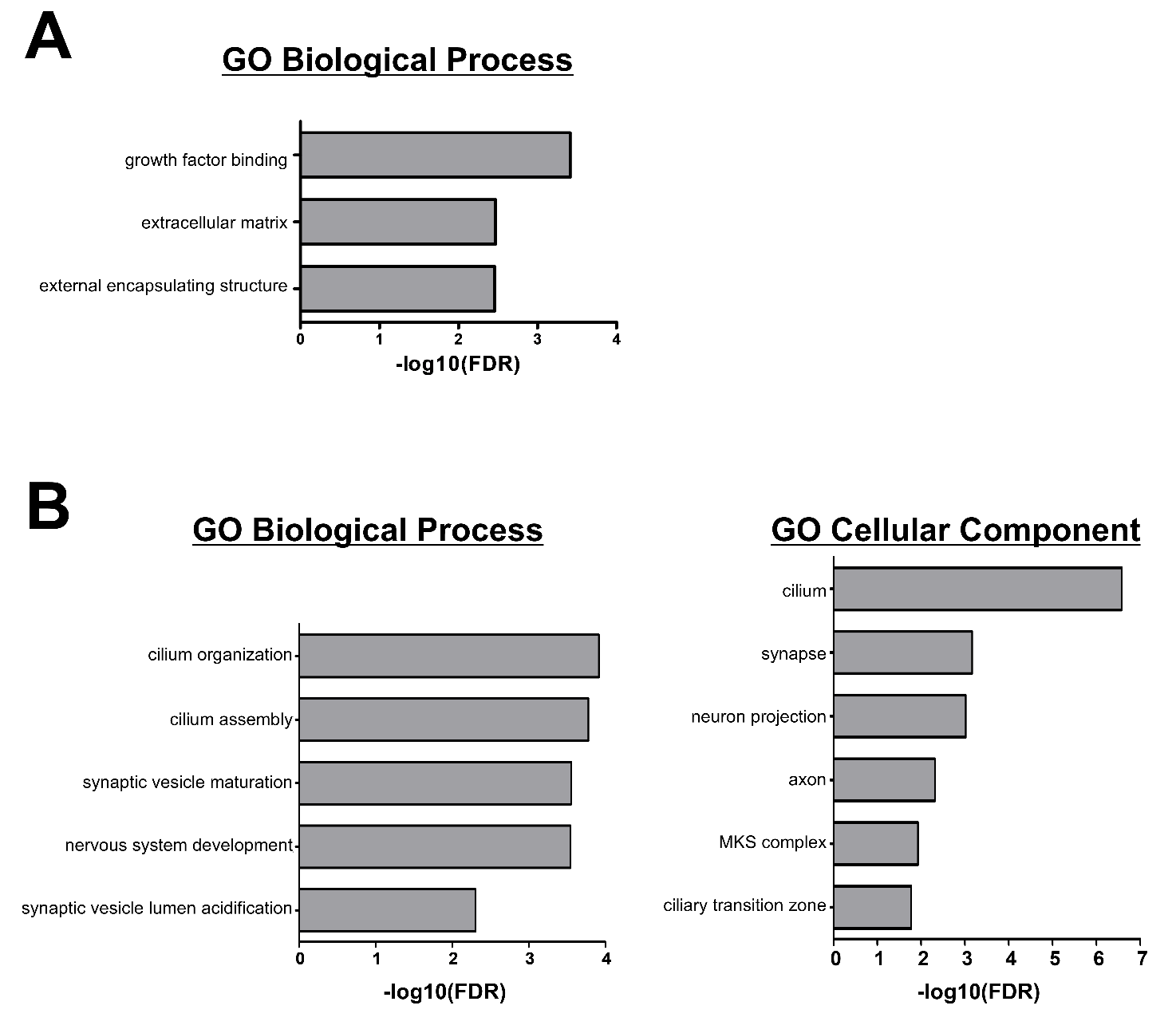


**Fig. S1 Gene Ontology Analysis of Genes Significantly Upregulated at FDR < 0.05**

The bar graph shows GO terms enriched among genes significantly upregulated in the CNROE cell line compared with the eGFP cell line (FDR < 0.05) (A). The bar graph shows GO terms enriched among genes that were significantly upregulated in the CNROE_PDM compared with eGFP (FDR < 0.05) (B).


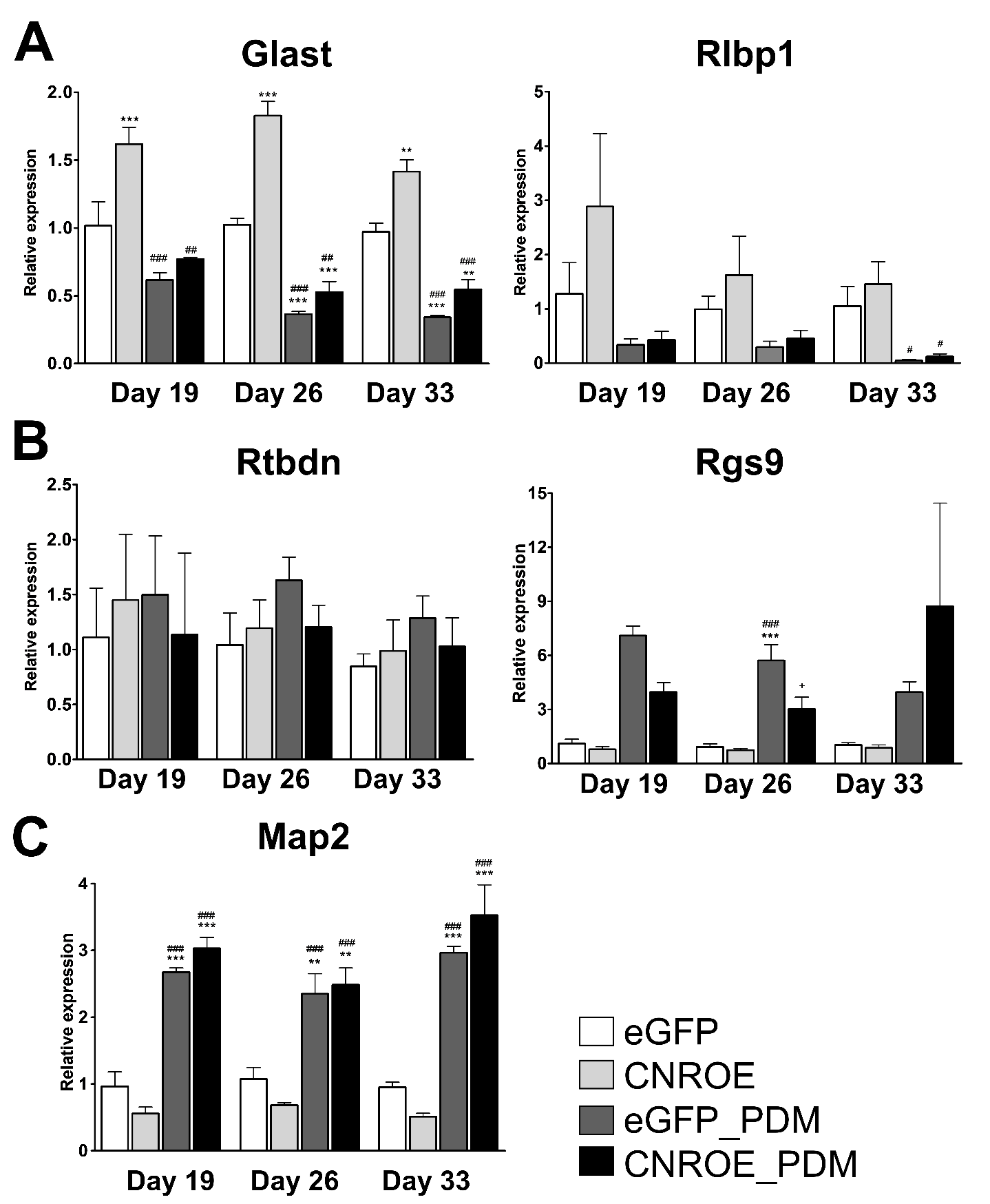


**Fig. S2 Time-course analysis of marker gene expression during differentiation.**

Gene expression was analyzed at 2–4 weeks (Day 19, 26, 33 respectively). Expression levels of Müller glia, photoreceptor, and neuronal markers were assessed. Data are presented as mean ± SE values. Statistical analysis was performed using Tukey’s multiple comparison test. (**, **, *** p < 0.05, 0.01, 0.001* compared to eGFP, *#, ##, ### p < 0.05, 0.01, 0.001* compared to CNROE, *+ p < 0.05* compared to eGFP_PDM. CNROE_PDM at 2 and 3 weeks: n=3; all other conditions: n = 4.
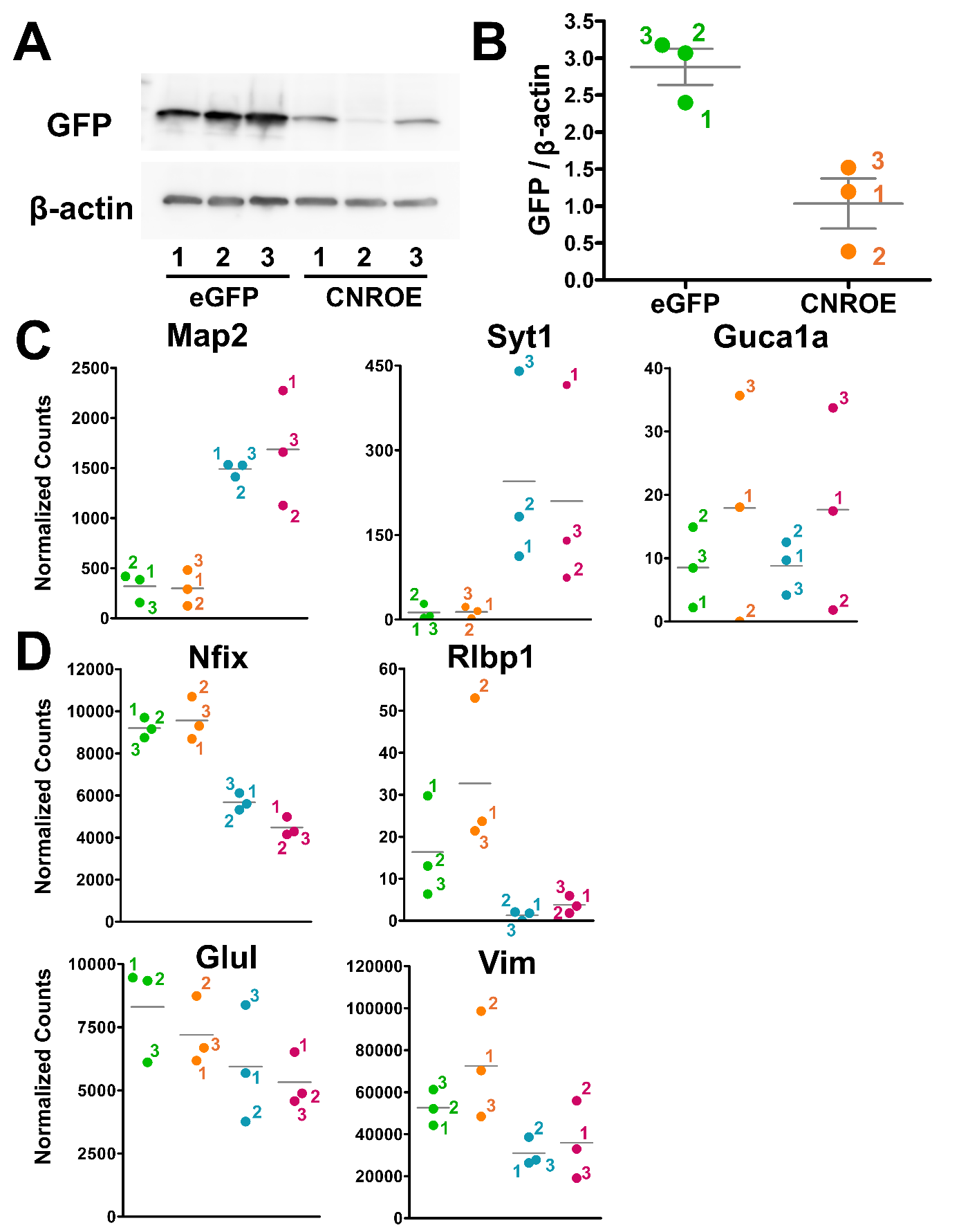


**Fig. S3 Inter-clonal Variability in Transcription Factor Expression and Differentiation Efficiency.**

Western blot analysis of eGFP expression in CNROE and eGFP clones. (A). Quantification of eGFP expression levels based on band intensities normalized to β-actin (B). Dot plots of normalized count data for neuronal- and photoreceptor-related genes (C) and Müller glia marker (D). In each clone, showing differences in expression levels across clones.


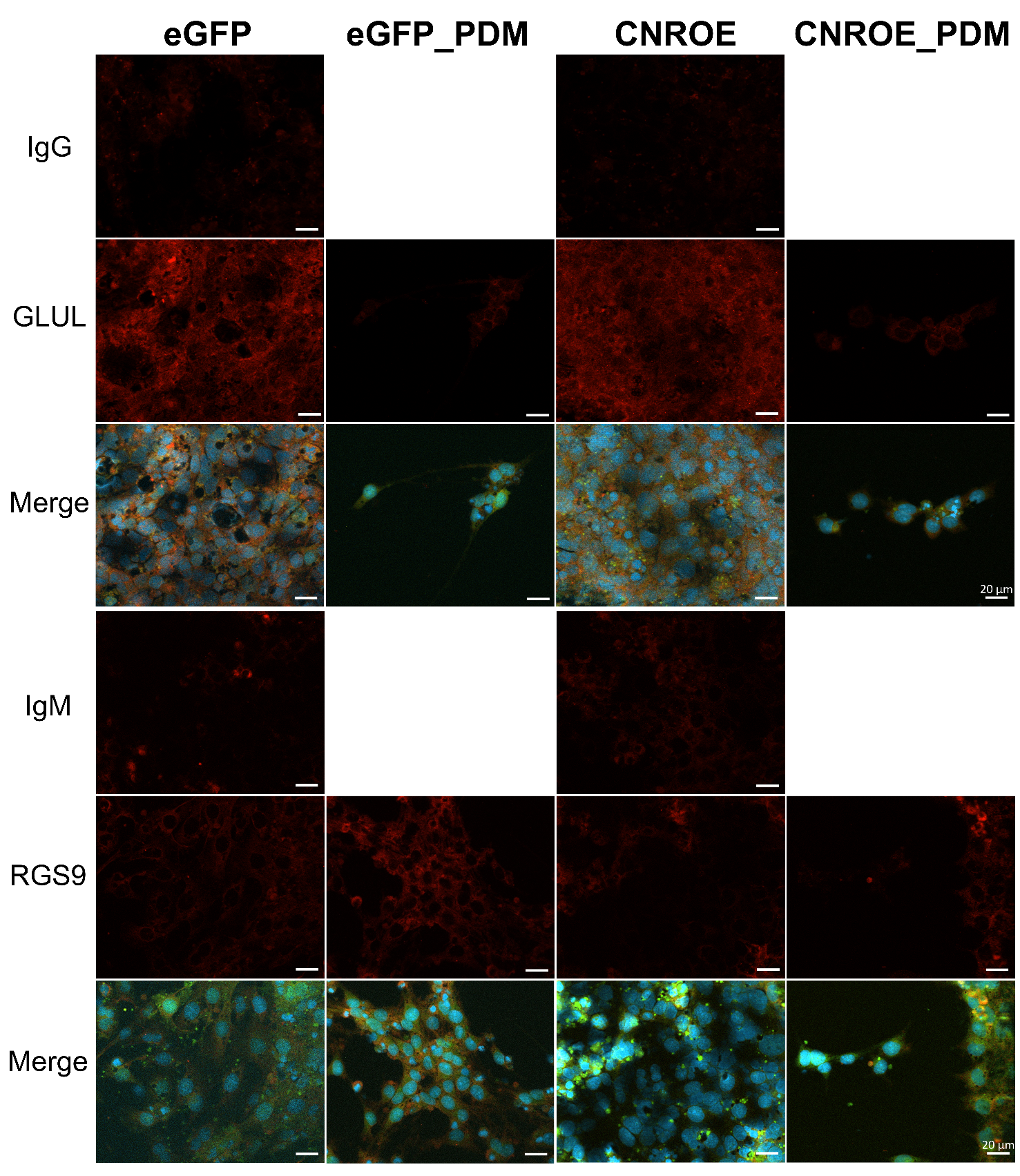


**Fig. S4 Immunocytochemistry**

Immunocytochemistry of the Müller glia marker GLUL and photoreceptor marker RGS9. Scale bar, 20 µm.

**
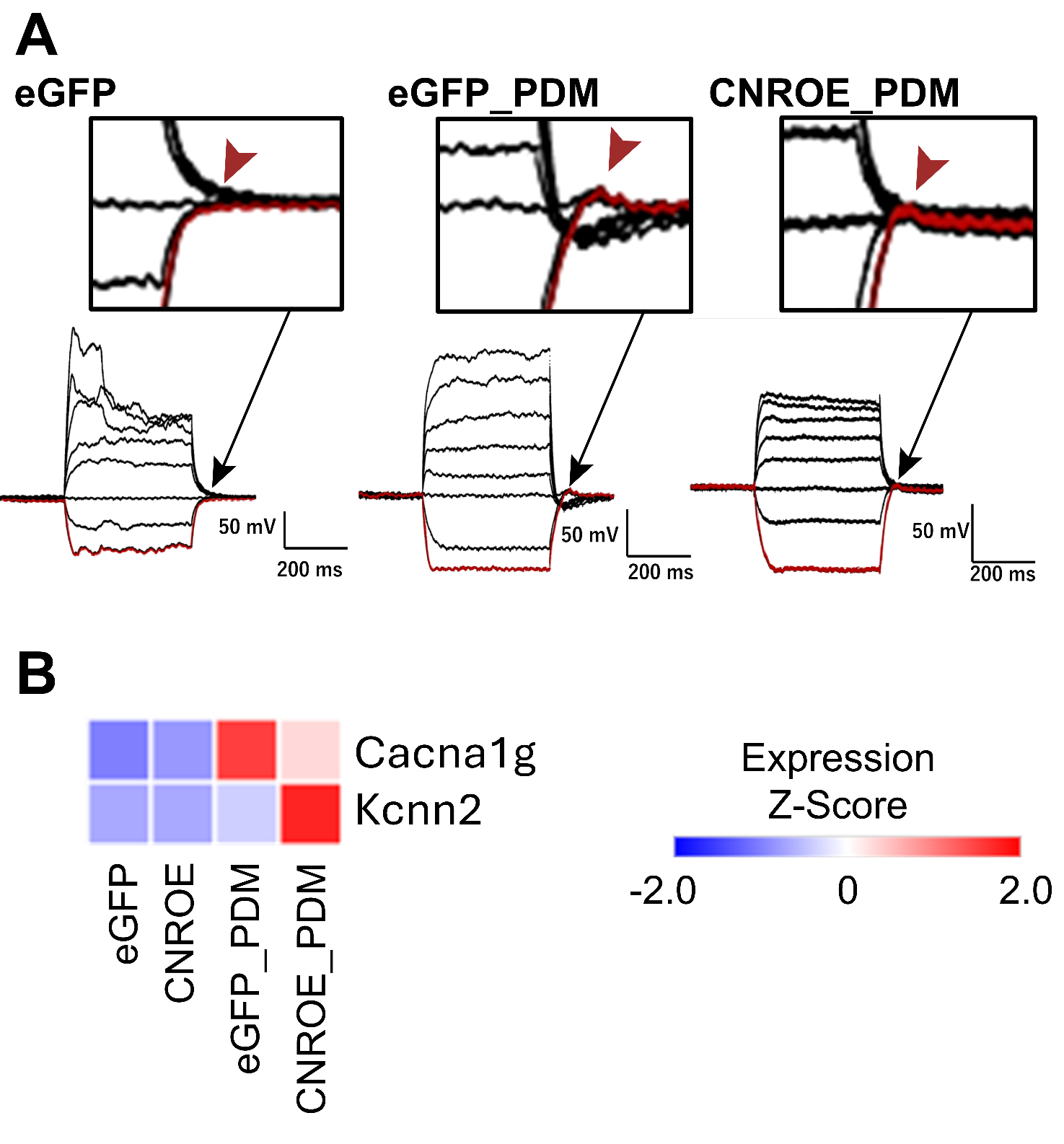
**

**Fig. S5 Electrophysiological Properties**

Representative voltage responses to hyperpolarizing current steps (A). Heatmap showing the expression of ion channels associated with rebound firing in each condition (B)

**
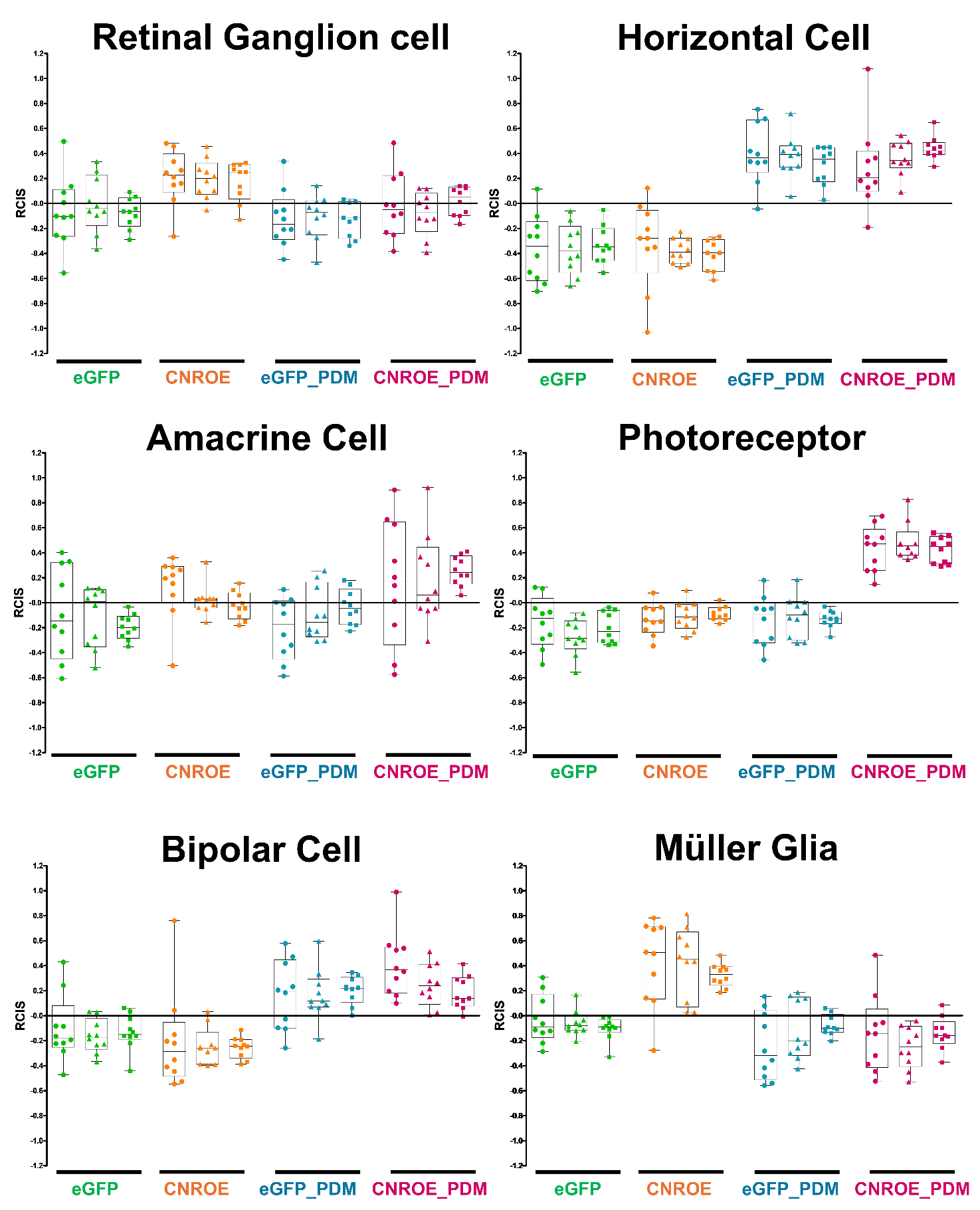
**

**Fig. S6 Sensitivity Analysis of RCIS across Different Marker Gene Subsets for Each Cell Type.**

Box plots show the results of a sensitivity analysis of Relative Cell Identity Score (RCIS) across retinal cell types. Each dot represents an RCIS value calculated from a randomly sampled subset of marker genes (n = 10 per condition). Colors and symbols indicate the percentage of genes sampled.(●:30%, ▲:50%, ■:70%)

| **Sample Name** | **Sample ID** | **Treatment Conditions** | **BioProject ID** | **BioSample ID** | **SRA Project ID** | **SRA Run ID** |
| --- | --- | --- | --- | --- | --- | --- |
| CNROE_1 | CNROE_line1 | CNROE, PDM(-) | PRJNA1295714 | SAMN50184331 | SRP605169 | SRR34792825 |
| CNROE_2 | CNROE_line2 | CNROE, PDM(-) | PRJNA1295714 | SAMN50184332 | SRP605169 | SRR34792824 |
| CNROE_3 | CNROE_line3 | CNROE, PDM(-) | PRJNA1295714 | SAMN50184333 | SRP605169 | SRR34792823 |
| eGFP_1 | EGFP_line1 | eGFP, PDM(-) | PRJNA1295714 | SAMN50184328 | SRP605169 | SRR34792830 |
| eGFP_2 | EGFP_line2 | eGFP, PDM(-) | PRJNA1295714 | SAMN50184329 | SRP605169 | SRR34792829 |
| eGFP_3 | EGFP_line3 | eGFP, PDM(-) | PRJNA1295714 | SAMN50184330 | SRP605169 | SRR34792826 |
| CNROEPDM_1 | CNROE_line1D | CNROE, PDM(+) | PRJNA1295714 | SAMN50184337 | SRP605169 | SRR34792819 |
| CNROEPDM_2 | CNROE_line2D | CNROE, PDM(+) | PRJNA1295714 | SAMN50184338 | SRP605169 | SRR34792828 |
| CNROEPDM_3 | CNROE_line3D | CNROE, PDM(+) | PRJNA1295714 | SAMN50184339 | SRP605169 | SRR34792827 |
| eGFPPDM_1 | EGFP_line1D | eGFP, PDM(+) | PRJNA1295714 | SAMN50184334 | SRP605169 | SRR34792822 |
| eGFPPDM_2 | EGFP_line2D | eGFP, PDM(+) | PRJNA1295714 | SAMN50184335 | SRP605169 | SRR34792821 |
| eGFPPDM_3 | EGFP_line3D | eGFP, PDM(+) | PRJNA1295714 | SAMN50184336 | SRP605169 | SRR34792820 |

**Table. S1 List of Accession IDs for BioProject, BioSample, and SRA**

| **Cell Type** | **Gene Symbol** | **Entrez ID** | **Reference** |
| --- | --- | --- | --- |
| Retinal ganglion cell | Atoh7 | 365564 | (Hoshino et al., 2017; Telegina, Kozhevnikova, Antonenko, & Kolosova, 2021) |
|  | Gap43 | 29423 | (Hoshino et al., 2017; Kim et al., 2016) |
|  | Pou4f2 | 171355 | (Hoshino et al., 2017; Rheaume et al., 2018; Telegina et al., 2021) |
|  | Pou4f1 | 114503 | (Hoshino et al., 2017; Rheaume et al., 2018) |
|  | Isl1 | 64444 | (Hoshino et al., 2017; Kim et al., 2016; Rheaume et al., 2018) |
|  | Tubb3 | 246118 | (Rheaume et al., 2018) |
|  | Map1b | 29456 | (Mack, Koester, & Pollerberg, 2000) |
|  | Ina | 24503 | (Kim et al., 2016) |
|  | Rbfox1 | 302920 | (Lei Gu, Joseph Caprioli, & Natik Piri, 2018) |
|  | Rbfox2 | 362950 | (Lei Gu et al., 2018) |
|  | Rbfox3 | 287847 | (Lin, Kuo, Chen, & Huang, 2018) |
|  | Sncg | 64347 | (Kim et al., 2016; Mahato et al., 2020) |
|  | Thy1 | 24832 | (Mahato et al., 2020) |
|  | Nefl | 83613 | (Mahato et al., 2020) |
|  | Nefm | 24588 | (Mahato et al., 2020) |
|  | Rbpms | 498642 | (Rheaume et al., 2018) |
|  | Shh | 29499 | (Hoshino et al., 2017) |
|  | Dlx1 | 296500 | (Hoshino et al., 2017; Rheaume et al., 2018) |
|  | Dlx2 | 296499 | (Hoshino et al., 2017; Rheaume et al., 2018) |
|  | Stmn2 | 84510 | (Laboissonniere et al., 2019) |
|  | Nrn1 | 83834 | (Janssen, Mac Nair, Dietz, Schlamp, & Nickells, 2013) |
| Horizontal cell | Ptf1a | 117034 | (Kim et al., 2016; Telegina et al., 2021) |
|  | Onecut1 | 25231 | (Hoshino et al., 2017) |
|  | Onecut2 | 307376 | (Hoshino et al., 2017) |
|  | Foxn4 | 288736 | (Kim et al., 2016; Telegina et al., 2021) |
|  | Lhx1 | 257634 | (Telegina et al., 2021) |
|  | Prox1 | 305066 | (Hoshino et al., 2017; Telegina et al., 2021) |
|  | Megf11 | 691517 | (Kay, Chu, & Sanes, 2012) |
|  | Calb1 | 83839 | (Hoshino et al., 2017) |
|  | Gja10 | 313126 | (Hombach et al., 2004) |
|  | Sall3 | 364910 | (Baba, Iida, & Watanabe, 2011) |
| Amacrine cell | Prox1 | 305066 | (Hoshino et al., 2017; Kim et al., 2016) |
|  | Ptf1a | 117034 | (Bassett & Wallace, 2012; Kim et al., 2016; Telegina et al., 2021) |
|  | Tfap2a | 306862 | (Hoshino et al., 2017; Kim et al., 2016) |
|  | Tfap2b | 301285 | (Cherry, Trimarchi, Stadler, & Cepko, 2009) |
|  | Foxn4 | 288736 | (Bassett & Wallace, 2012; Kim et al., 2016; Telegina et al., 2021) |
|  | Gad1 | 24379 | (Hoshino et al., 2017) |
|  | Gad2 | 24380 | (Hoshino et al., 2017) |
|  | Chat | 290567 | (Hoshino et al., 2017) |
|  | Slc32a1 | 83612 | (Kunzevitzky, Almeida, & Goldberg, 2010) |
|  | Slc6a9 | 116509 | (Kunzevitzky et al., 2010) |
|  | Calb2 | 117059 | (Kunzevitzky et al., 2010) |
|  | Sox2 | 499593 | (Cherry et al., 2009) |
| Bipolar cell | Otx2 | 305858 | (Telegina et al., 2021) |
|  | Vsx2 | 171360 | (Bassett & Wallace, 2012; Telegina et al., 2021) |
|  | Grm6 | 24419 | (Brooks et al., 2019; Hoshino et al., 2017; Kim et al., 2016) |
|  | Gng13 | 685451 | (Woods, Mountjoy, Muir, Ross, & Atan, 2018) |
|  | Trpm1 | 361586 | (Woods et al., 2018) |
|  | Bhlhe23 | 499952 | (Brooks et al., 2019) |
|  | Vsx1 | 689704 | (Bassett & Wallace, 2012; Brooks et al., 2019; Hoshino et al., 2017) |
|  | Lhx4 | 360858 | (Bassett & Wallace, 2012; Brooks et al., 2019; Hoshino et al., 2017) |
|  | Trnp1 | 688990 | (Brooks et al., 2019) |
|  | Cacna2d3 | 306243 | (Woods et al., 2018) |
|  | Car8 | 297814 | (Woods et al., 2018) |
|  | Pcp2 | 304195 | (Kim et al., 2016) |
|  | Prkca | 24680 | (Brooks et al., 2019; Hoshino et al., 2017; Kim et al., 2016) |
|  | Cabp5 | 365194 | (Brooks et al., 2019; Hoshino et al., 2017) |
|  | Bhlhe22 | 365748 | (Brooks et al., 2019; Hoshino et al., 2017) |
|  | Gabrr1 | 29694 | (Brooks et al., 2019) |
|  | Prdm8 | 305198 | (Hoshino et al., 2017) |
| Photoreceptor | Otx2 | 305858 | (Bassett & Wallace, 2012; Hoshino et al., 2017) |
|  | Crx | 60446 | (Hoshino et al., 2017; Kim et al., 2016) |
|  | Neurod1 | 29458 | (Bassett & Wallace, 2012; Kim et al., 2016) |
|  | Prdm1 | 309871 | (Bassett & Wallace, 2012) |
|  | Lhx4 | 360858 | (Buenaventura, Corseri, & Emerson, 2019) |
|  | Tbx2 | 303398 | (Hoshino et al., 2017) |
|  | Rax | 114213 | (Irie et al., 2015) |
|  | Ascl1 | 64186 | (Kim et al., 2016; Mahato et al., 2020) |
|  | Rcvrn | 140936 | (Brooks et al., 2019; Hoshino et al., 2017; Kim et al., 2016) |
|  | Rbp3 | 24711 | (Kim et al., 2016) |
|  | Guca1a | 301233 | (Kim et al., 2016; Mahato et al., 2020) |
|  | Rgs9 | 29481 | (Kaya et al., 2019) |
|  | Pdc | 25343 | (Osakada et al., 2008) |
|  | Nrl | 290221 | (Brooks et al., 2019; Hoshino et al., 2017; Kim et al., 2016; Mahato et al., 2020) |
|  | Nr2e3 | 100365683 | (Brooks et al., 2019; Hoshino et al., 2017; Kim et al., 2016; Mahato et al., 2020) |
|  | Pias3 | 83614 | (Bassett & Wallace, 2012) |
|  | Aipl1 | 59110 | (Kim et al., 2016) |
|  | Gnat1 | 363143 | (Brooks et al., 2019; Kim et al., 2016; Mahato et al., 2020) |
|  | Pde6b | 289878 | (Brooks et al., 2019; Kim et al., 2016) |
|  | Pde6g | 688297 | (Kim et al., 2016) |
|  | Rtbdn | 304667 | (Kaya et al., 2019) |
|  | Rpgrip1 | 305850 | (Osakada et al., 2008) |
|  | Rp1 | 681377 | (Kaya et al., 2019; Kim et al., 2016) |
|  | Cngb1 | 83686 | (Brooks et al., 2019; Kim et al., 2016; Mahato et al., 2020) |
|  | Grk1 | 81760 | (Kaya et al., 2019; Kim et al., 2016) |
|  | Sag | 25539 | (Brooks et al., 2019; Kim et al., 2016) |
|  | Slc24a2 | 84550 | (Kaya et al., 2019) |
|  | Rho | 24717 | (Brooks et al., 2019; Hoshino et al., 2017; Kim et al., 2016; Mahato et al., 2020) |
|  | Thrb | 24831 | (Bassett & Wallace, 2012; Kim et al., 2016; Mahato et al., 2020) |
|  | Rxrg | 83574 | (Bassett & Wallace, 2012; Kim et al., 2016) |
|  | Gngt2 | 690825 | (Bassett & Wallace, 2012; Kim et al., 2016) |
|  | Gnat2 | 365901 | (Brooks et al., 2019; Hoshino et al., 2017; Kim et al., 2016) |
|  | Pde6c | 361752 | (Brooks et al., 2019; Kim et al., 2016) |
|  | Pde6h | 114248 | (Brooks et al., 2019; Hoshino et al., 2017; Kim et al., 2016) |
|  | Cngb3 | 500418 | (Brooks et al., 2019; Kaya et al., 2019; Kim et al., 2016) |
|  | Arr3 | 171107 | (Brooks et al., 2019; Kaya et al., 2019; Kim et al., 2016) |
|  | Opn1sw | 81644 | (Brooks et al., 2019; Kim et al., 2016) |
|  | Opn1mw | 89810 | (Brooks et al., 2019; Hoshino et al., 2017; Kim et al., 2016) |
| Müller glia | Sox2 | 499593 | (Hoshino et al., 2017; Kim et al., 2016) |
|  | Sox9 | 140586 | (Kim et al., 2016) |
|  | Notch1 | 25496 | (Kim et al., 2016) |
|  | Hes1 | 29577 | (Bassett & Wallace, 2012; Kim et al., 2016) |
|  | Hes5 | 79225 | (Bassett & Wallace, 2012; Kim et al., 2016) |
|  | Nfib | 29227 | (Kim et al., 2016) |
|  | Nfix | 81524 | (Kim et al., 2016) |
|  | Vim | 81818 | (Brooks et al., 2019; Hoshino et al., 2017; Kim et al., 2016) |
|  | Rlbp1 | 293049 | (Hoshino et al., 2017; Kim et al., 2016) |
|  | Dkk3 | 171548 | (Kim et al., 2016) |
|  | Cntfr | 313173 | (Kim et al., 2016) |
|  | Dbi | 25045 | (Kim et al., 2016) |
|  | Aqp4 | 25293 | (Mahato et al., 2020) |
|  | Glul | 24957 | (Hoshino et al., 2017; Kim et al., 2016) |
|  | Slc1a3 | 29483 | (Hoshino et al., 2017) |
|  | Kcnj10 | 29718 | (Hoshino et al., 2017) |
|  | Gfap | 24387 | (Dahl, 1979) |
|  | Clu | 24854 | (Mahato et al., 2020) |
|  | Ca2 | 54231 | (Hoshino et al., 2017) |

**Table S2 Retinal cell-specific marker genes and transcription factors used in heatmap plotting and calculating the Relative Cell Identity Score (RCIS)**

**【Table S2 Reference】**

Baba, Y., Iida, A., & Watanabe, S. (2011). Sall3 plays essential roles in horizontal cell maturation through regulation of neurofilament expression levels. Biochimie, 93(6), 1037–1046. doi:10.1016/j.biochi.2011.02.016

Bassett, E. A., & Wallace, V. A. (2012). Cell fate determination in the vertebrate retina. Trends in Neurosciences, 35(9), 565–573. doi:10.1016/j.tins.2012.05.004

Brooks, M. J., Chen, H. Y., Kelley, R. A., Mondal, A. K., Nagashima, K., De Val, N., … Swaroop, A. (2019). Improved Retinal Organoid Differentiation by Modulating Signaling Pathways Revealed by Comparative Transcriptome Analyses with Development In Vivo. Stem Cell Reports, 13(5), 891–905. doi:10.1016/j.stemcr.2019.09.009

Buenaventura, D. F., Corseri, A., & Emerson, M. M. (2019). Identification of Genes With Enriched Expression in Early Developing Mouse Cone Photoreceptors. Investigative Opthalmology & Visual Science, 60(8), 2787. doi:10.1167/iovs.19-26951

Cherry, T. J., Trimarchi, J. M., Stadler, M. B., & Cepko, C. L. (2009). Development and diversification of retinal amacrine interneurons at single cell resolution. Proceedings of the National Academy of Sciences, 106(23), 9495–9500. doi:10.1073/pnas.0903264106

Dahl, D. (1979). The radial glia of Müller in the rat retina and their response to injury. An immunofluorescence study with antibodies to the glial fibrillary acidic (GFA) protein. Experimental Eye Research, 28(1), 63–69. doi:10.1016/0014-4835(79)90106-4

Hombach, S., Janssen‐Bienhold, U., Söhl, G., Schubert, T., Büssow, H., Ott, T., … Willecke, K. (2004). Functional expression of connexin57 in horizontal cells of the mouse retina. European Journal of Neuroscience, 19(10), 2633–2640. doi:10.1111/j.0953-816X.2004.03360.x

Hoshino, A., Ratnapriya, R., Brooks, M. J., Chaitankar, V., Wilken, M. S., Zhang, C., … Reh, T. A. (2017). Molecular Anatomy of the Developing Human Retina. Developmental Cell, 43(6), 763-779.e4. doi:10.1016/j.devcel.2017.10.029

Irie, S., Sanuki, R., Muranishi, Y., Kato, K., Chaya, T., & Furukawa, T. (2015). Rax Homeoprotein Regulates Photoreceptor Cell Maturation and Survival in Association with Crx in the Postnatal Mouse Retina. Molecular and Cellular Biology, 35(15), 2583–2596. doi:10.1128/MCB.00048-15

Janssen, K. T., Mac Nair, C. E., Dietz, J. A., Schlamp, C. L., & Nickells, R. W. (2013). Nuclear Atrophy of Retinal Ganglion Cells Precedes the Bax -Dependent Stage of Apoptosis. Investigative Opthalmology & Visual Science, 54(3), 1805. doi:10.1167/iovs.11-9310

Kay, J. N., Chu, M. W., & Sanes, J. R. (2012). MEGF10 and MEGF11 mediate homotypic interactions required for mosaic spacing of retinal neurons. Nature, 483(7390), 465–469. doi:10.1038/nature10877

Kaya, K. D., Chen, H. Y., Brooks, M. J., Kelley, R. A., Shimada, H., Nagashima, K., … Swaroop, A. (2019). Transcriptome-based molecular staging of human stem cell-derived retinal organoids uncovers accelerated photoreceptor differentiation by 9-cis retinal. Molecular Vision, 25, 663–678.

Kim, J. W., Yang, H. J., Brooks, M. J., Zelinger, L., Karakülah, G., Gotoh, N., … Swaroop, A. (2016). NRL-Regulated Transcriptome Dynamics of Developing Rod Photoreceptors. Cell Reports, 17(9), 2460–2473. doi:10.1016/j.celrep.2016.10.074

Kunzevitzky, N. J., Almeida, M. V, & Goldberg, J. L. (2010). Amacrine cell gene expression and survival signaling: differences from neighboring retinal ganglion cells. Investigative Ophthalmology & Visual Science, 51(7), 3800–3812. doi:10.1167/iovs.09-4540

Laboissonniere, L. A., Goetz, J. J., Martin, G. M., Bi, R., Lund, T. J. S., Ellson, L., … Trimarchi, J. M. (2019). Molecular signatures of retinal ganglion cells revealed through single cell profiling. Scientific Reports, 9(1), 15778. doi:10.1038/s41598-019-52215-4

Lei Gu, Joseph Caprioli, & Natik Piri. (2018). Temporal and spatial expression patterns of Rbfox1 and Rbfox2 during mouse retinal development. In Investigative Ophthalmology & Visual Science. Honolulu, Hawaii.

Lin, Y.-S., Kuo, K.-T., Chen, S.-K., & Huang, H.-S. (2018). RBFOX3/NeuN is dispensable for visual function. PLOS ONE, 13(2), e0192355. doi:10.1371/journal.pone.0192355

Mack, T. G. A., Koester, M. P., & Pollerberg, G. E. (2000). The Microtubule-Associated Protein MAP1B Is Involved in Local Stabilization of Turning Growth Cones. Molecular and Cellular Neuroscience, 15(1), 51–65. doi:10.1006/mcne.1999.0802

Mahato, B., Kaya, K. D., Fan, Y., Sumien, N., Shetty, R. A., Zhang, W., … Chavala, S. H. (2020). Pharmacologic fibroblast reprogramming into photoreceptors restores vision. Nature, 581(7806), 83–88. doi:10.1038/s41586-020-2201-4

Osakada, F., Ikeda, H., Mandai, M., Wataya, T., Watanabe, K., Yoshimura, N., … Takahashi, M. (2008). Toward the generation of rod and cone photoreceptors from mouse, monkey and human embryonic stem cells. Nature Biotechnology, 26(2), 215–224. doi:10.1038/nbt1384

Rheaume, B. A., Jereen, A., Bolisetty, M., Sajid, M. S., Yang, Y., Renna, K., … Trakhtenberg, E. F. (2018). Single cell transcriptome profiling of retinal ganglion cells identifies cellular subtypes. Nature Communications, 9(1), 2759. doi:10.1038/s41467-018-05134-3

Telegina, D. V, Kozhevnikova, O. S., Antonenko, A. K., & Kolosova, N. G. (2021). Features of Retinal Neurogenesis as a Key Factor of Age-Related Neurodegeneration: Myth or Reality? International Journal of Molecular Sciences, 22(14). doi:10.3390/ijms22147373

Woods, S. M., Mountjoy, E., Muir, D., Ross, S. E., & Atan, D. (2018). A comparative analysis of rod bipolar cell transcriptomes identifies novel genes implicated in night vision. Scientific Reports, 8(1), 5506. doi:10.1038/s41598-018-23901-6

| **Sample ID** | **Total Sequences** | **% GC** | **Avg Read Length** | **Adapter Contamination** |
| --- | --- | --- | --- | --- |
| EGFP_line1_1.fq | 20764657 | 48 | 150 | PASS |
| EGFP_line1_2.fq | 20764657 | 49 | 150 | PASS |
| EGFP_line2_1.fq | 23698911 | 48 | 150 | PASS |
| EGFP_line2_2.fq | 23698911 | 49 | 150 | PASS |
| EGFP_line3_1.fq | 21041746 | 48 | 150 | PASS |
| EGFP_line3_2.fq | 21041746 | 49 | 150 | PASS |
| EGFP_line1D_1.fq | 22688421 | 49 | variable(31-150) | PASS |
| EGFP_line1D_2.fq | 22688421 | 50 | variable(31-150) | PASS |
| EGFP_line2D_1.fq | 24738849 | 49 | variable(31-150) | PASS |
| EGFP_line2D_2.fq | 24738849 | 50 | variable(31-150) | PASS |
| EGFP_line3D_1.fq | 22512738 | 49 | 150 | PASS |
| EGFP_line3D_2.fq | 22512738 | 50 | 150 | PASS |
| CNROE_line1_1.fq | 19875264 | 48 | 150 | PASS |
| CNROE_line1_2.fq | 19875264 | 49 | 150 | PASS |
| CNROE_line2_1.fq | 19660231 | 49 | 150 | PASS |
| CNROE_line2_2.fq | 19660231 | 49 | 150 | PASS |
| CNROE_line3_1.fq | 24710758 | 48 | 150 | PASS |
| CNROE_line3_2.fq | 24710758 | 49 | 150 | PASS |
| CNROE_line1D_1.fq | 25206166 | 49 | variable(31-150) | PASS |
| CNROE_line1D_2.fq | 25206166 | 50 | variable(31-150) | PASS |
| CNROE_line2D_1.fq | 24468968 | 49 | 150 | PASS |
| CNROE_line2D_2.fq | 24468968 | 49 | 150 | PASS |
| CNROE_line3D_1.fq | 20959285 | 49 | variable(31-150) | PASS |
| CNROE_line3D_2.fq | 20959285 | 50 | variable(31-150) | PASS |

**Table. S3 Summary of FastQC Results for Each Sample**

| **Sample ID** | **Total Reads** | **Uniquely Mapped (%)** | **Multi-mapped (%)** | **Unmapped (%)** | **Overall Mapping Rate (%)** |
| --- | --- | --- | --- | --- | --- |
| EGFP_line1 | 20764657 | 92.56 | 2.11 | 5.29 | 94.71 |
| EGFP_line2 | 24738849 | 87.53 | 2.07 | 10.23 | 89.77 |
| EGFP_line3 | 21041746 | 93.10 | 2.24 | 4.57 | 95.43 |
| EGFP_line1D | 20263113 | 89.31 | 1.9 | 8.66 | 91.34 |
| EGFP_line2D | 21654408 | 87.53 | 2.07 | 10.23 | 89.77 |
| EGFP_line3D | 19788374 | 87.9 | 1.9 | 10.11 | 89.89 |
| CNROE_line1 | 18493348 | 93.05 | 2.09 | 4.78 | 95.22 |
| CNROE_line2 | 19660231 | 93.23 | 2.01 | 4.70 | 95.30 |
| CNROE_line3 | 24710758 | 92.29 | 2.13 | 5.51 | 94.49 |
| CNROE_line1D | 25206166 | 88.54 | 1.83 | 9.50 | 90.50 |
| CNROE_line2D | 24468968 | 88.21 | 1.76 | 9.96 | 90.04 |
| CNROE_line3D | 20959285 | 87.89 | 2.07 | 9.89 | 90.11 |

**Table. S4 Summary of STAR Alignment Results for Each Sample**

| **Sample ID** | **Assingned Reads** | **％ Assingned** | **Unassingned Multimapping** | **Unassingned  No Feature** | **Unassingned Ambiguous** |
| --- | --- | --- | --- | --- | --- |
| EGFP_line1 | 32634535 | 80.39 | 2159066 | 4807004 | 996983 |
| EGFP_line2 | 36683798 | 79.34 | 2425776 | 6006865 | 1120331 |
| EGFP_line3 | 33101824 | 79.63 | 2386066 | 5047952 | 1031762 |
| EGFP_line1D | 34555242 | 80.74 | 2270630 | 4728855 | 1242129 |
| EGFP_line2D | 37375826 | 81.11 | 2771318 | 4520290 | 1412700 |
| EGFP_line3D | 33932825 | 81.14 | 2245330 | 4374939 | 1268984 |
| CNROE_line1 | 30938140 | 79.17 | 2089464 | 5078620 | 969936 |
| CNROE_line2 | 30944554 | 80.14 | 1956622 | 4760070 | 953606 |
| CNROE_line3 | 38480996 | 79.75 | 2641016 | 5940853 | 1187305 |
| CNROE_line1D | 37854563 | 80.42 | 2433960 | 5433992 | 1347509 |
| CNROE_line2D | 36941296 | 81.45 | 2185894 | 4852980 | 1374744 |
| CNROE_line3D | 31642369 | 80.81 | 2314814 | 4100081 | 1098058 |

**Table. S5 Summary of featureCounts Quantification Results for Each Sample**

| **Retinal ganglion cell** |  | EGFP | | | CNROE | | | EGFP_PDM | | | CNROE_PDM | | |
| --- | --- | --- | --- | --- | --- | --- | --- | --- | --- | --- | --- | --- | --- |
|  | Extraction ratio | 30 % | 50 % | 70 % | 30 % | 50 % | 70 % | 30 % | 50 % | 70 % | 30 % | 50 % | 70 % |
|  | extracted genes | 7 | 11 | 16 | 7 | 11 | 16 | 7 | 11 | 16 | 7 | 11 | 16 |
|  | mean | -0.068 | -0.006 | -0.075 | 0.207 | 0.195 | 0.179 | -0.124 | -0.108 | -0.121 | -0.015 | -0.082 | 0.017 |
|  | Standard Deviation | 0.281 | 0.220 | 0.120 | 0.215 | 0.153 | 0.156 | 0.224 | 0.176 | 0.141 | 0.260 | 0.172 | 0.116 |
|  | Coefficient of Variation | 4.151 | 39.167 | 1.601 | 1.040 | 0.786 | 0.872 | 1.801 | 1.641 | 1.166 | 17.392 | 2.094 | 6.724 |
| **Horizontal cell** |  | EGFP | | | CNROE | | | EGFP_PDM | | | CNROE_PDM | | |
|  | Extraction ratio | 30 % | 50 % | 70 % | 30 % | 50 % | 70 % | 30 % | 50 % | 70 % | 30 % | 50 % | 70 % |
|  | extracted genes | 5 | 8 | 11 | 5 | 8 | 11 | 5 | 8 | 11 | 5 | 8 | 11 |
|  | mean | -0.361 | -0.363 | -0.333 | -0.325 | -0.377 | -0.410 | 0.402 | 0.377 | 0.303 | 0.284 | 0.363 | 0.440 |
|  | Standard Deviation | 0.265 | 0.197 | 0.148 | 0.342 | 0.098 | 0.122 | 0.243 | 0.170 | 0.149 | 0.333 | 0.135 | 0.093 |
|  | Coefficient of Variation | 0.735 | 0.542 | 0.445 | 1.051 | 0.259 | 0.299 | 0.605 | 0.450 | 0.492 | 1.173 | 0.371 | 0.211 |
| **Amacrine cell** |  | EGFP | | | CNROE | | | EGFP_PDM | | | CNROE_PDM | | |
|  | Extraction ratio | 30 % | 50 % | 70 % | 30 % | 50 % | 70 % | 30 % | 50 % | 70 % | 30 % | 50 % | 70 % |
|  | extracted genes | 4 | 6 | 9 | 4 | 6 | 9 | 4 | 6 | 9 | 4 | 6 | 9 |
|  | mean | -0.084 | -0.109 | -0.194 | 0.127 | 0.023 | -0.025 | -0.206 | -0.092 | -0.036 | 0.163 | 0.178 | 0.254 |
|  | Standard Deviation | 0.365 | 0.241 | 0.099 | 0.253 | 0.123 | 0.108 | 0.245 | 0.212 | 0.138 | 0.491 | 0.357 | 0.118 |
|  | Coefficient of Variation | 4.348 | 2.207 | 0.510 | 2.001 | 5.402 | 4.400 | 1.193 | 2.303 | 3.888 | 3.014 | 2.005 | 0.462 |
| **Photoreceptor** |  | EGFP | | | CNROE | | | EGFP_PDM | | | CNROE_PDM | | |
|  | Extraction ratio | 30 % | 50 % | 70 % | 30 % | 50 % | 70 % | 30 % | 50 % | 70 % | 30 % | 50 % | 70 % |
|  | extracted genes | 6 | 9 | 12 | 6 | 9 | 12 | 6 | 9 | 12 | 6 | 9 | 12 |
|  | mean | -0.155 | -0.274 | -0.201 | -0.133 | -0.103 | -0.089 | -0.145 | -0.107 | -0.132 | 0.432 | 0.484 | 0.422 |
|  | Standard Deviation | 0.201 | 0.141 | 0.124 | 0.124 | 0.113 | 0.056 | 0.197 | 0.164 | 0.068 | 0.179 | 0.149 | 0.104 |
|  | Coefficient of Variation | 1.298 | 0.517 | 0.619 | 0.936 | 1.096 | 0.625 | 1.361 | 1.524 | 0.514 | 0.414 | 0.307 | 0.247 |
| **Bipolar cell** |  | EGFP | | | CNROE | | | EGFP_PDM | | | CNROE_PDM | | |
|  | Extraction ratio | 30 % | 50 % | 70 % | 30 % | 50 % | 70 % | 30 % | 50 % | 70 % | 30 % | 50 % | 70 % |
|  | extracted genes | 12 | 19 | 27 | 12 | 19 | 27 | 12 | 19 | 27 | 12 | 19 | 27 |
|  | mean | -0.105 | -0.164 | -0.133 | -0.205 | -0.246 | -0.255 | 0.160 | 0.164 | 0.205 | 0.410 | 0.247 | 0.183 |
|  | Standard Deviation | 0.261 | 0.131 | 0.143 | 0.385 | 0.145 | 0.084 | 0.277 | 0.204 | 0.110 | 0.260 | 0.166 | 0.131 |
|  | Coefficient of Variation | 2.478 | 0.796 | 1.074 | 1.876 | 0.591 | 0.329 | 1.731 | 1.243 | 0.535 | 0.635 | 0.672 | 0.720 |
| **Müller glia** |  | EGFP | | | CNROE | | | EGFP_PDM | | | CNROE_PDM | | |
|  | Extraction ratio | 30 % | 50 % | 70 % | 30 % | 50 % | 70 % | 30 % | 50 % | 70 % | 30 % | 50 % | 70 % |
|  | extracted genes | 7 | 12 | 17 | 7 | 12 | 17 | 7 | 12 | 17 | 7 | 12 | 17 |
|  | mean | -0.030 | -0.060 | -0.104 | 0.422 | 0.422 | 0.324 | -0.248 | -0.111 | -0.080 | -0.144 | -0.251 | -0.141 |
|  | Standard Deviation | 0.191 | 0.101 | 0.093 | 0.341 | 0.282 | 0.091 | 0.267 | 0.237 | 0.082 | 0.303 | 0.165 | 0.127 |
|  | Coefficient of Variation | 6.306 | 1.687 | 0.891 | 0.808 | 0.669 | 0.280 | 1.077 | 2.129 | 1.025 | 2.106 | 0.656 | 0.905 |

**Table. S6 Summary of the Sensitivity Analysis of RCIS across Different Marker Gene Subsets for Each Retinal Cell Type**
